# Integrated Assessment of Heavy Metals in Mining-Affected Soils in China: Source Identification, Ecological Risks, and Sustainable Remediation

**DOI:** 10.3390/toxics14080689

**Published:** 2026-08-04

**Authors:** Yuanchao Zhao, Jin Hua, Tingting Fan, Yan Zhou, Xiang Wang, Shengtian Zhang, Qun Li

**Affiliations:** Nanjing Institute of Environmental Science, Ministry of Ecology and Environment, Nanjing 210042, China; zhaoyuanchao@nies.org (Y.Z.);

**Keywords:** metal mining areas, soil pollution, source apportionment, source-oriented risk assessment, ecological risk assessment, material–biological synergistic remediation, triple bottom line, sustainable remediation

## Abstract

China is the world’s largest producer and consumer of mineral resources. While large-scale development of metal mineral resources has supported rapid national economic development, it has also caused serious heavy metal pollution in soils in mining areas. This paper systematically reviews the latest research progress on heavy metal pollution in soils in mining areas, focusing on Chinese metal mining areas, covering pollution characteristics, source identification, risk assessment, and sustainable remediation technologies. Meta-analysis shows that smelting sources contribute more than 50% of soil Cd in southwestern Pb-Zn mining areas, making them priority targets for risk management. This review aims to provide theoretical support and decision-making references for precise source tracing, scientific assessment, and green remediation of heavy metal pollution affecting soils in Chinese metal mining areas.

## 1. Introduction

As the world’s largest producer and consumer of mineral resources, the development and utilization of metal minerals in China is an important material foundation for rapid national economic development [1]. However, long-term mining and smelting activities have not only consumed large amounts of land resources but also released significant amounts of heavy metals (HMs) into surrounding soils through tailings accumulation, acid mine drainage, and atmospheric deposition [2,3,4]. Due to the non-degradability, bioaccumulation, and high toxicity of HMs, soil pollution in Chinese metal mining areas has become a prominent regional environmental issue, seriously threatening ecological security and human health [5]. Based on the “National Soil Pollution Situation Investigation Bulletin” issued by the Ministry of Environmental Protection and the Ministry of Land and Resources in 2014, among the 70 soil sampling sites, 33.4% of the sites exceeded the standard. Specifically, the soil surrounding non-ferrous metal mining areas suffered relatively severe pollution from cadmium, arsenic, lead, etc., and environmental issues affecting soils in abandoned industrial and mining land were quite prominent [6,7]. For example, in Pb-Zn mining areas in western China, toxic HMs like Cd and Pb in the soil migrate from underground to the ground through plants or grains, forming serious “chemical timebombs” [8,9]. Such pollution not only causes deterioration of the soil’s physical and chemical properties, decline in biodiversity, crop yield reduction, or even complete crop failure, but more seriously, HMs can enter the food chain through pathways such as soil–crop–human or soil–dust–human [10,11]. Epidemiological studies show that in the vicinity of some smelters, blood lead levels in children are significantly higher than the normal concentration limits, which seriously affects the development of the nervous system [12]. However, despite extensive research on individual aspects of mining soil pollution, significant knowledge gaps remain. Existing studies have primarily focused on investigating pollution status and qualitative description of risk [5], while lacking a comprehensive synthesis that integrates pollution characteristics, source identification, risk assessment, and remediation technologies. Moreover, previous reviews often present a fragmented dualistic structure between pollution characteristics–risk assessment and remediation technologies, failing to establish a complete logical chain from risk identification to risk management [6]. Most importantly, the China-specific challenges, such as the dual superposition of natural geological high background values and long-term anthropogenic disturbance in southwestern karst regions, have not been adequately addressed by existing international frameworks [7,8]. Therefore, conducting comprehensive assessment and remediation research on heavy metal pollution affecting soils in mining area, with a focus on China-specific challenges, has important practical significance and academic value.

**Figure 1:**
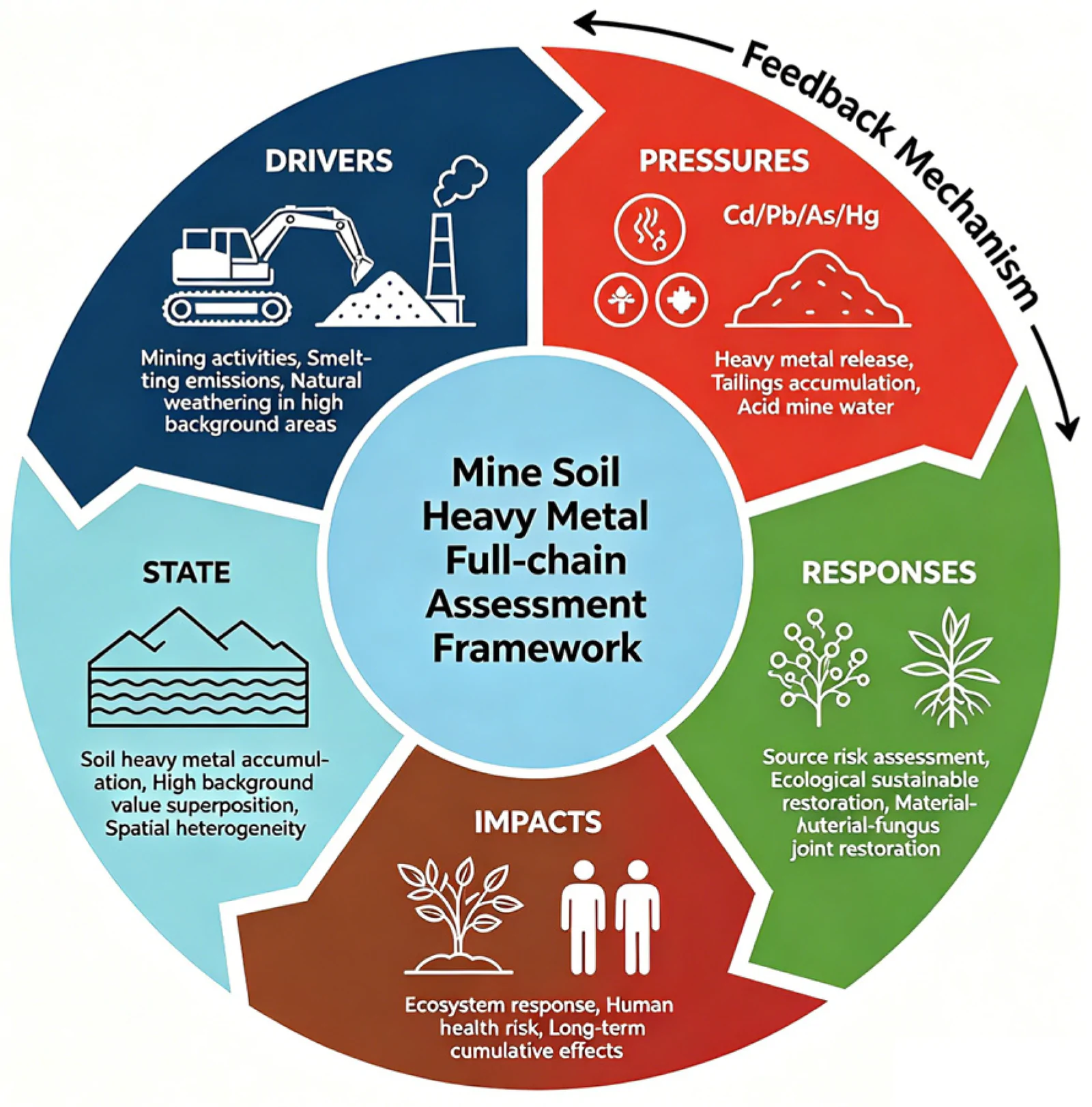
DPSIR (Driving Forces–Pressures–State–Impacts–Responses) framework for comprehensive assessment of soil HMs in Chinese metal mining areas. This framework encompasses five core components: (1) DRIVERS, including mining activities, smelting emissions, and natural weathering; (2) PRESSURES, involving heavy metal release, tailings accumulation, and acid mine drainage generation; (3) STATE, reflecting soil heavy metal accumulation, high geochemical background superposition, and spatial heterogeneity; (4) IMPACTS, including ecosystem responses, human health risks, and long-term accumulation effects; (5) RESPONSES, covering source-oriented risk assessment, sustainable remediation technologies, and material–biological synergistic strategies. The components form a closed loop through feedback mechanisms, providing theoretical support for systematic assessment and management of soil HMs in mining areas.

Traditional research on the soil environment in mining areas has mostly focused on investigation of pollution status and qualitative description of risk. With the advancement of analytical techniques and the development of data science, current research is undergoing a transformation from descriptive to quantitative and from single assessment to systematic management. In terms of identifying sources of pollution, receptor models such as positive matrix factorization (PMF) and absolute principal component scores–multiple linear regression (APCS-MLR) have been widely applied [13,14]. However, single models have limitations in handling complex nonlinear systems. The introduction of machine learning (e.g., self-organizing maps, SOMs) for multi-source data fusion analysis has become an important direction for future methodological development in source identification [15,16,17]. In risk assessment, deterministic evaluation methods based on total content are being gradually replaced by assessment models based on bioavailable fraction and probability distribution [18,19]. More notably, the establishment of the “Source-Oriented Risk Assessment” framework has provided a promising approach for direct quantitative attribution from “pollution sources” to “risk contributions” [20,21,22,23]. With regard to remediation technology, physicochemical remediation techniques (such as solidification/stabilization and soil washing) are effective but limited by high energy consumption and secondary pollution risks [24,25]. Green technologies like phytoremediation and microbial remediation are favored for their environmental friendliness but face challenges associated with long remediation cycles and low efficiency [26,27,28,29]. Therefore, combining environmental functional materials such as biochar and nanomaterials with bioremediation to construct a multi-phase synergistic remediation system of materials–plants–microorganisms has become a research hotspot in current green remediation technology development [30,31,32]. Despite numerous reviews focusing on heavy metal pollution remediation in mining areas, most studies present a fragmented dualistic structure between pollution characteristics–risk assessment and remediation technologies, failing to establish a complete logical chain from risk identification to risk management [33,34,35]. Given the particularity and urgency of soil pollution control in Chinese mining areas, it is particularly necessary to systematically define out the full-chain methodological system of source identification–risk assessment–remediation technology. Against this background, integrating the “Source-Oriented Risk Assessment” framework and establishing quantitative correlations between pollution sources, risk contributions, and priority control will provide theoretical support for solving the current management dilemma of who is responsible for pollution (Figure 1).

This study aims to systematically review the latest research progress on heavy metal pollution in Chinese metal mining area soils and construct a comprehensive analytical framework covering pollution characteristics, source identification, risk assessment, and remediation technologies. By comparing pollution differences among different mining area types (non-ferrous metal mines, coal mines) in China and analyzing source tracing difficulties in high-background-value areas such as southwestern China, integrating probabilistic risk and source–risk coupling assessment methods, this paper also focuses on reviewing sustainable remediation technologies represented by biochar and PGPR microbial agents. It introduces the “triple bottom line” principle for multi-dimensional assessment, providing theoretical references and decision support for precise management and green remediation of soil pollution in Chinese metal mining areas.

## 2. Methodology

### 2.1. Literature Search Strategy

This systematic review strictly follows the PRISMA guidelines for literature search and screening (Figure S1). The search strategy was systematically constructed to ensure comprehensive coverage of relevant research in the field of heavy metal pollution in Chinese mining area soils. The search covered four core databases, taking into account both Chinese and English literature resources: Web of Science Core Collection (4495 papers), Scopus (140 papers), CNKI (46 papers), and Wanfang (20 papers), totaling 4701 papers initially retrieved. The search time range was set from 1 January 2005 to 1 June 2026. Quality assessment was conducted using the AMSTAR 2 tool for systematic reviews and the NOS scale for observational studies, ensuring only high-quality studies were included in the final analysis.

English terms were mainly used to search the Web of Science Core Collection and Scopus databases, covering heavy metal-related terms (heavy metal, metal contamination, potentially toxic element, PTE), mining area-related terms (mining soil, mining area, mine site, tailing, mine waste, coal mining, lead–zinc mine, copper mine, gold mine, rare earth mine), geographic qualifiers (China, Chinese), and research topic terms (source apportionment, source identification, risk assessment, ecological risk, health risk, remediation, phytoremediation). Boolean operators (AND, OR, NOT) were used to combine search terms, with field tags (TI, AB, KY) applied to improve search precision.

### 2.2. Literature Screening Criteria and Process

Sources that met the following criteria were included in this study. The research area was metal mining regions within China, including non-ferrous metal mines, coal mines, gold mines, rare earth mines, etc.; research topics covered at least one item from among soil heavy metal pollution characteristics, source apportionment, risk assessment, or remediation technologies; research types included peer-reviewed original research papers, high-quality review articles, meta-analyses, and published doctoral/master dissertations; the language was Chinese or English; and the sources provided extractable quantitative or qualitative data. Literature sources meeting any of the following criteria were excluded: research area outside China; a focus only on water, atmosphere, or biological tissues without involving soil; pure laboratory simulation studies without actual field application data; the full text could not be obtained or key data were missing; duplicate publications with the same DOI or highly similar titles; conference abstracts, short communications, and low-quality review articles.

The literature search and screening process followed a structured workflow adapted from PRISMA guidelines. The initial database search yielded 4701 records: Web of Science Core Collection (*n* = 4495), Scopus (*n* = 140), CNKI (*n* = 46), and Wanfang (*n* = 20). After removing 115 duplicates, 4586 records underwent regional screening, of which 385 were excluded for geographic irrelevance, leaving 4201 records. Title and abstract screening excluded 3 irrelevant papers, leaving 4198 papers. A comprehensive scoring ranking was then applied to select 300 priority reading candidates based on three weighted criteria: relevance (50%), journal impact factor (30%), and citation count (20%). The composite score was calculated as follows: Composite score = (Relevance score × 0.5) + (IF score × 0.3) + (Citation score × 0.2). A total of 300 candidate papers were selected for priority reading.

Full-text screening of the 300 priority candidates involved two independent reviewers applying standardized inclusion/exclusion criteria and quality assessment tools: AMSTAR 2 (for reviews/meta-analyses, requiring ≥6/16) and NOS (for original research, requiring ≥6/9). This process excluded 112 papers due to geographic irrelevance (*n* = 28), thematic irrelevance (*n* = 35), data limitations (*n* = 27), or quality concerns (*n* = 22), resulting in 188 studies retained for qualitative synthesis. Discrepancies between reviewers were resolved through discussion or arbitration by a third researcher, with Cohen’s kappa coefficient of 0.85 indicating excellent inter-rater reliability. The PRISMA flowchart illustrating this screening process is shown in Figure S1. Detailed information about the 152 references is cited in Supplementary Table S1.

## 3. Status and Characteristics of HMs Pollution in Mining Area Soils

The development and utilization of metal mineral resources have promoted economic development while causing serious soil heavy metal pollution problems. Unlike ordinary urban or agricultural pollution sources, soil pollution in metal mining areas is characterized by high intensity, long history, and high complexity. Mining activities not only directly release HMs through tailings accumulation and mine water discharge, but also change regional topography and hydrogeological conditions, accelerating the diffusion and activation of HMs. This summarizes the typical characteristics of heavy metal pollution in Chinese metal mining area soils from two dimensions: spatial distribution pattern and characteristic occurrence form.

### 3.1. Spatial Distribution and Migration Patterns

**Figure 2:**
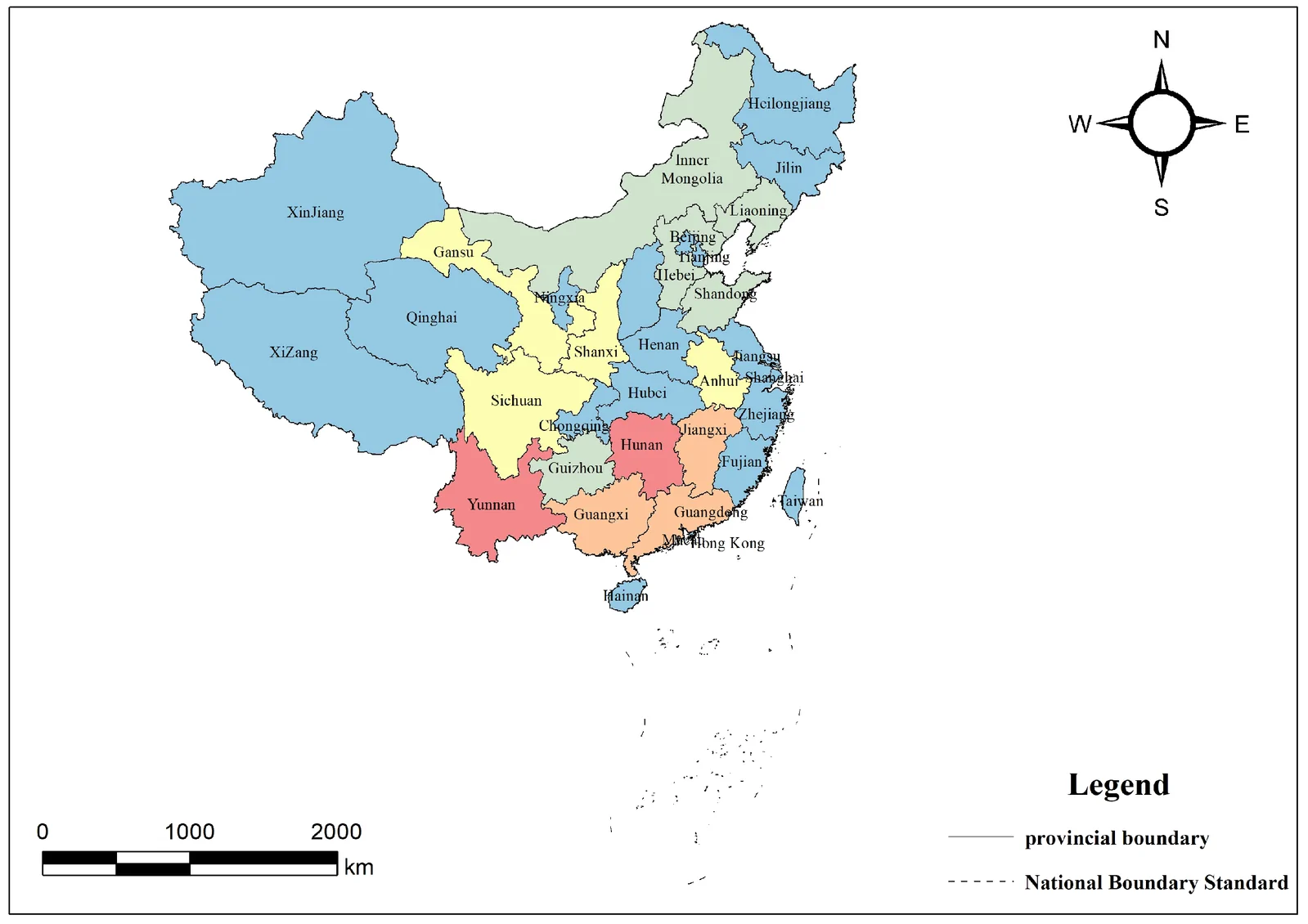
Distribution map of research hotspots in non-ferrous metal mining areas in China in the past 20 years. The color gradient from blue to red indicates the number of research hotspots in each province, with blue representing a low number of research hotspots and red representing a high number of research hotspots.

The spatial distribution of HMs in metal mining area soils is extremely uneven, often showing island-like or strip-like distribution centered on pollution sources [36,37]. The degree of pollution shows a significant negative correlation with the distance from pollution sources, but this attenuation relationship is strongly modulated by dominant wind direction, terrain slope, and surface runoff direction [38,39,40,41]. In recent years, combining geographic information system (GIS) data with geostatistical methods such as ordinary kriging, and inverse distance weighting (IDW), researchers have been able to visually display the spatial variation characteristics of HMs at the mining area scale [42,43]. A summary analysis of typical metal mining areas was extracted from the screened literature (Figure 2). The results show that soil heavy metal pollution in China’s metal mining areas exhibits significant regional aggregation, and research hotspots are mainly concentrated in southwestern China.

Different types of metal mining areas show significant differences in heavy metal enrichment types and pollution degrees due to differences in ore-forming geological background and smelting processes [44,45]. Pb-Zn mining areas are heavily polluted by soil HMs. Studies on typical Pb-Zn mining areas in Hunan, Yunnan, and other places show that the contents of Cd, Pb, Zn, Cu, and other elements in surrounding soils are usually several to dozens of times the screening values for agricultural land. Especially around smelters, due to long-term atmospheric deposition, surface soils often form significant Cd-Pb combined pollution zones [46,47,48,49]. For example, in the study of the Pb-Zn smelting area in Shanxi, it was found that within 8 km of the smelter, the average Cd content in soil exceeded the risk control value [50]. The pollution migration pattern in such areas usually shows radial diffusion centered on the smelting chimney, with pollution levels significantly higher in downwind areas than in upwind areas [51]. Cu (Mo) mining areas are another type of area typically at risk of high pollution. A significant feature of these mining areas is the production of large amounts of strongly acidic mine water (acid mine drainage, AMD). Taking Dexing Copper Mine in Jiangxi as an example, with its long mining history and huge scale, acidic water produced by oxidation of tailings ponds and waste rock piles can significantly activate sulfide minerals in ores, causing large amounts of HMs such as Cu, Cd, Mo, and Pb to be released and migrate with surface runoff. Therefore, pollution in copper mining areas often shows “acid-driven” characteristics, with extremely strong migration ability of HMs, easily causing serious acidification and heavy metals exceeding standards in regional surface water and groundwater [2,52]. Gold mining (processing) areas and their surroundings often face risk of combined cyanide (CN^−^) and HMs pollution. During heap leaching and cyanidation gold extraction processes, cyanide-containing waste liquid is produced along with highly toxic elements such as As, Hg, and Sb associated with ores, which are released in large quantities into the environment. Especially in some mining areas, due to the lack of effective anti-seepage measures, the combined pollution of cyanide and As poses a great threat to soil microbial communities and groundwater safety [53,54,55]. Polymetallic/rare metal mining areas (such as ionic rare earth mines in southern China) face unique pollution challenges. Ammonium salts (such as ammonium sulfate) are widely used as leaching agents during mining, causing serious ammonia nitrogen pollution and soil compaction in mining areas and surrounding soils [56,57]. More importantly, natural radionuclides (such as thorium, uranium) and HMs (such as Pb, Cd) are often associated with the ore-forming processes of rare earth mines [58,59]. Therefore, pollution assessment in such mining areas must consider not only the ecological toxicity of HMs but also the long-term health risks posed by radioactive pollution. Metal smelting areas, unlike primary mining areas, are usually near densely populated areas or transportation hubs, with longer pollution diffusion distances and more direct impact on people [60,61]. With increasing distance, the occurrence forms of HMs also show regular evolution. Near the smelting area, they are mainly water-soluble and exchangeable forms with high activity, while in long-distance diffusion areas, they gradually transform into more stable residual forms [62,63,64]. This feature indicates that fine-grained HMs from smelting emissions have strong long-distance migration capabilities, and their bioavailability changes dynamically during migration [63,64,65].

### 3.2. Speciation and Bioavailability

The environmental behavior and ecological toxicity of HMs in soil do not depend on their total amount, but largely depend on their speciation. HMs in soil usually exist in different forms such as water-soluble, exchangeable, carbonate-bound, Fe-Mn oxide-bound, organic-bound, and residual forms. Among these, the first three (especially exchangeable) are considered biologically available active fractions, while the residual form is fixed in mineral lattices and difficult for organisms to utilize [66,67,68].

#### 3.2.1. Speciation Classification and Environmental Indicative Significance

Sequential extraction methods (such as the Tessier five-step extraction method and BCR three-step extraction method of the European Community Bureau of Reference) are currently the mainstream methods for studying heavy metal speciation and bioavailability. Although the classification standards are slightly different, their core idea is consistent: simulating the release potential of HMs under different environmental conditions through extractants of different strengths [69,70,71,72]. Numerous studies on soils in mining areas show that HMs from anthropogenic sources (such as Pb and Zn from smelting emissions) mainly exist in non-residual forms (active forms), which is the fundamental reason for the extremely high risk of polluted soils in mining areas. The speciation distribution of heavy metals in soils across different functional areas of a zinc smelting site has been systematically characterized. The results show that Zn and Cd in smelting zone soils mainly exist in acid-extractable and reducible forms, which are extremely sensitive to changes in environmental pH. Research reveals that Fe-Mn oxide-bound forms are important “sinks” for HMs in mining area soils. When soils are under flooded reducing conditions (such as paddy fields) or when organic matter decomposes in large quantities, these bound HMs are reactivated and released, thus forming “secondary pollution” sources [73,74].

#### 3.2.2. Aging Effects and Transformation Mechanisms

**Figure 3:**
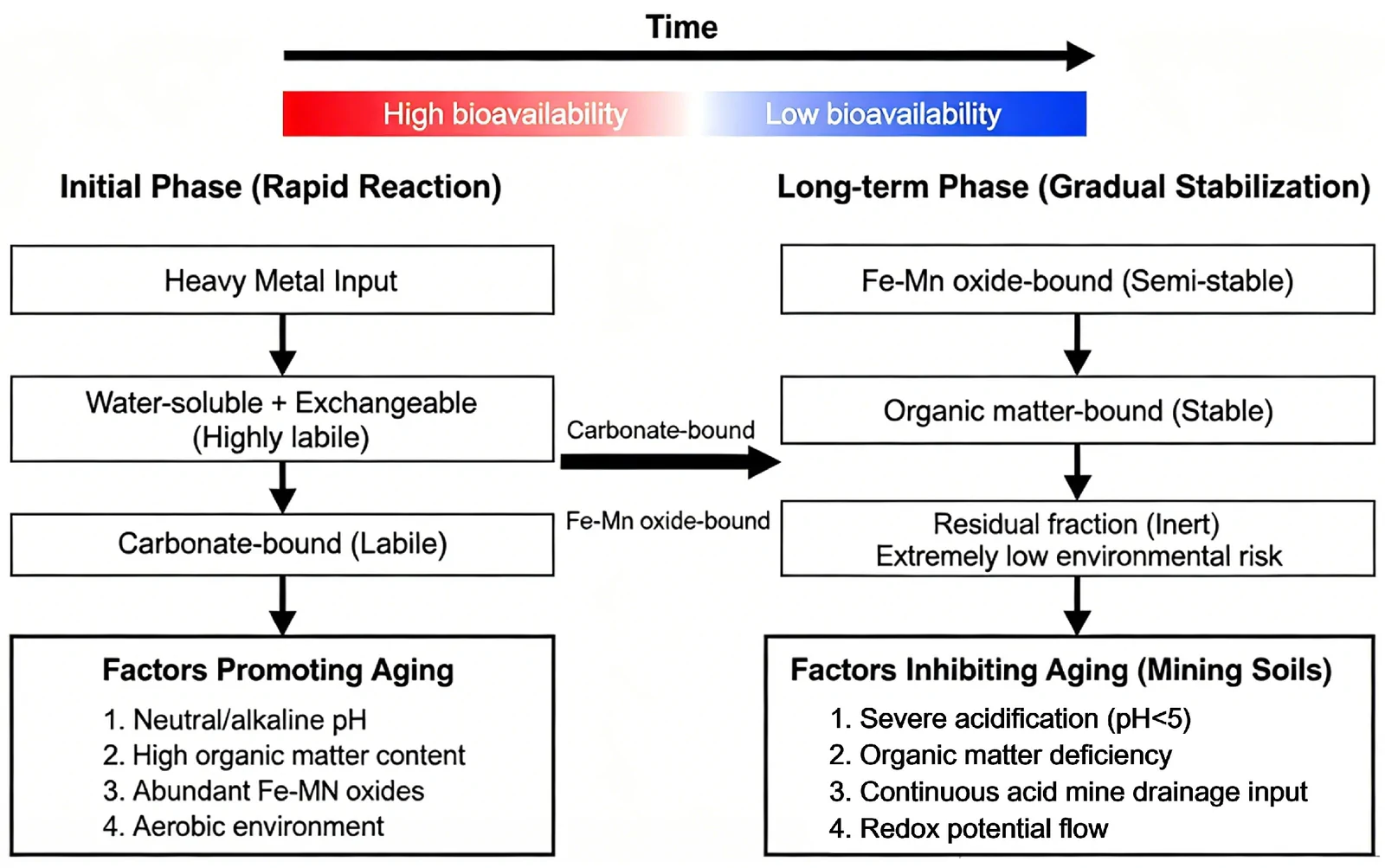
Schematic illustration of heavy metal speciation and bioavailability transformation in mining soils.

Aging of HMs in soil refers to the natural process by which exogenous HMs (Figure 3), after entering the soil, undergo a series of physical and chemical reactions such as adsorption, precipitation, and complexation over time, transforming from highly bioavailable fractions to less bioavailable fractions, ultimately leading to a gradual decrease in their bioavailability [75,76,77]. Theoretically, long-term natural aging can effectively reduce the environmental risk of HMs. However, the prevalent problems of severe acidification and organic matter deficiency in mining-affected soils have greatly limited the rate and extent of natural aging [78,79,80]. Numerous studies have demonstrated that heavy metal aging in soil exhibits distinct phase characteristics. In the initial phase after heavy metal input, the speciation transformation rate is fast and the activity decreases significantly; after entering the long-term phase, the transformation rate slows down markedly, and some bioavailable heavy metal fractions can remain stable even for decades [81,82,83,84]. Notably, the aging process in mining soils is far from a simple unidirectional stabilization process. The continuous input of acid mine drainage (AMD) and mineral processing reagents triggers the secondary oxidation of minerals such as pyrite, a process with dual effects. On the one hand, the H^+^ produced by oxidation further lowers soil pH, causing the re-dissolution of immobilized HMs. On the other hand, the newly formed amorphous iron oxides exert strong adsorption and encapsulation effects on HMs. Furthermore, elevated temperatures in the context of global climate change accelerate the decomposition of soil organic matter and alter the partition coefficients of HMs among different binding phases, potentially re-mobilizing originally stable HMs. This makes the long-term environmental risk of sequestered HMs in mining soils more complex and uncertain [85,86,87,88,89].

### 3.3. Regional-Specific Synthesis

Significant spatial heterogeneity exists in heavy metal pollution patterns across different regions of China. In the southwestern karst areas (Yunnan, Guizhou, Guangxi), the combination of high geological background values and intensive mining activities creates a unique “natural–anthropogenic coupling” pollution pattern, where Cd and Pb contents often exceed national soil quality standards by 50–100 times [3,4]. The northwestern mining regions (Shaanxi, Gansu) are characterized by copper and molybdenum mining, with AMD-induced acidification accelerating heavy metal mobilization, resulting in Cu and Cd concentrations 10–50 times the background values [5]. In contrast, eastern industrial zones (Zhejiang, Jiangsu) exhibit mixed pollution sources including mining, smelting, and industrial discharge, with relatively lower but more widespread heavy metal accumulation [6]. The northeastern coal mining areas show distinct Hg and As pollution from coal combustion and gangue stacking [7]. This regional differentiation highlights the need for targeted management strategies that account for local geological conditions and anthropogenic activities.

## 4. Source Identification and Quantitative Contribution Analysis

Accurate identification of soil heavy metal pollution sources is a prerequisite for targeted remediation and precise management. Soil pollution around mining areas often shows the characteristics of double superposition of high natural geological background values and anthropogenic mining activity inputs. Single qualitative analysis can no longer meet the needs of refined management. In recent years, the introduction of receptor models and machine learning algorithms has enabled quantitative analysis and spatial visualization of pollution source contributions, greatly promoting the transformation of environmental science to data-driven.

### 4.1. Receptor Models: From Qualitative Identification to Quantitative Analysis

Traditional source apportionment mainly relies on multivariate statistical methods such as correlation analysis and principal component analysis (PCA). These methods can effectively identify potential pollution factors (such as industrial factors and traffic factors), but it is difficult to quantify the specific contribution percentage of each source to receptor sites, and misjudgments often occur when dealing with high-background-value areas [13,14]. The Positive Matrix Factorization (PMF) model has become the mainstream tool for soil heavy metal source apportionment due to its non-negative constraints and uncertainty estimation capabilities [2,22]. An optimized PC-PMF algorithm built on partition calculation was developed to enhance the robustness of conventional PMF models during the processing of large-scale and spatially heterogeneous regional datasets [90]. This method effectively reduces model residuals and improves the accuracy of source apportionment for HMs (such as Cd, Pb) in agricultural soils by first performing spatial clustering on the study area and then running PMF separately in sub-regions. Uncertainty analysis (bootstrap testing) must be performed when applying the PMF model, or the analysis results may have large deviations [91]. For complex scenarios such as mining areas, studies show that PMF can effectively separate independent sources such as tailings weathering and leaching, dry and wet atmospheric deposition, and agricultural activities, and calculate the contribution ratio of each source. For example, in typical southwestern Pb-Zn mining areas, mining and smelting sources usually contribute more than 50% of Cd in surface soils [92,93]. Despite the obvious advantages of PMF, the model does not provide standard source composition profiles, and the results have a certain degree of non-uniqueness. Therefore, more and more studies have begun to try combining PMF with technologies such as absolute principal component acore–multiple linear regression (APCS-MLR), and TEM-EDS to reduce the uncertainty of analysis results through multi-model verification (Table 1).

### 4.2. Machine Learning-Enabled Intelligent Source Tracing

In the study of source apportionment of HMs in mine soils, the fine distinction between natural and anthropogenic sources in high geological background areas has long been a core technical bottleneck and academic controversy. Machine learning algorithms, which excel at nonlinear fitting, provide an efficient technical path to solve this problem. Ensemble algorithms such as Random Forest (RF) and Gradient Boosting Tree (GBT) can quantify the relative contributions of intrinsic geological factors and extrinsic anthropogenic factors through feature importance ranking, and these have become the mainstream methods for quantitative source apportionment in the current traceability field [81,98,99]. Unsupervised neural networks such as self-organizing maps (SOMs) can accurately identify similar pollution patterns and intuitively present the spatial gradient distribution of pollution sources. They cross-validate with the Positive Matrix Factorization (PMF) model in mining scenarios and successfully delineate the core “high pollution-high risk” pollution sources around tailings ponds [97]. Multi-model fusion and machine learning–isotope coupling technologies are becoming the current research hotspots and important development directions for in-depth source apportionment of HMs in mining areas.

## 5. Management-Oriented Integrated Eco-Health Risk Assessment

**Figure 4:**
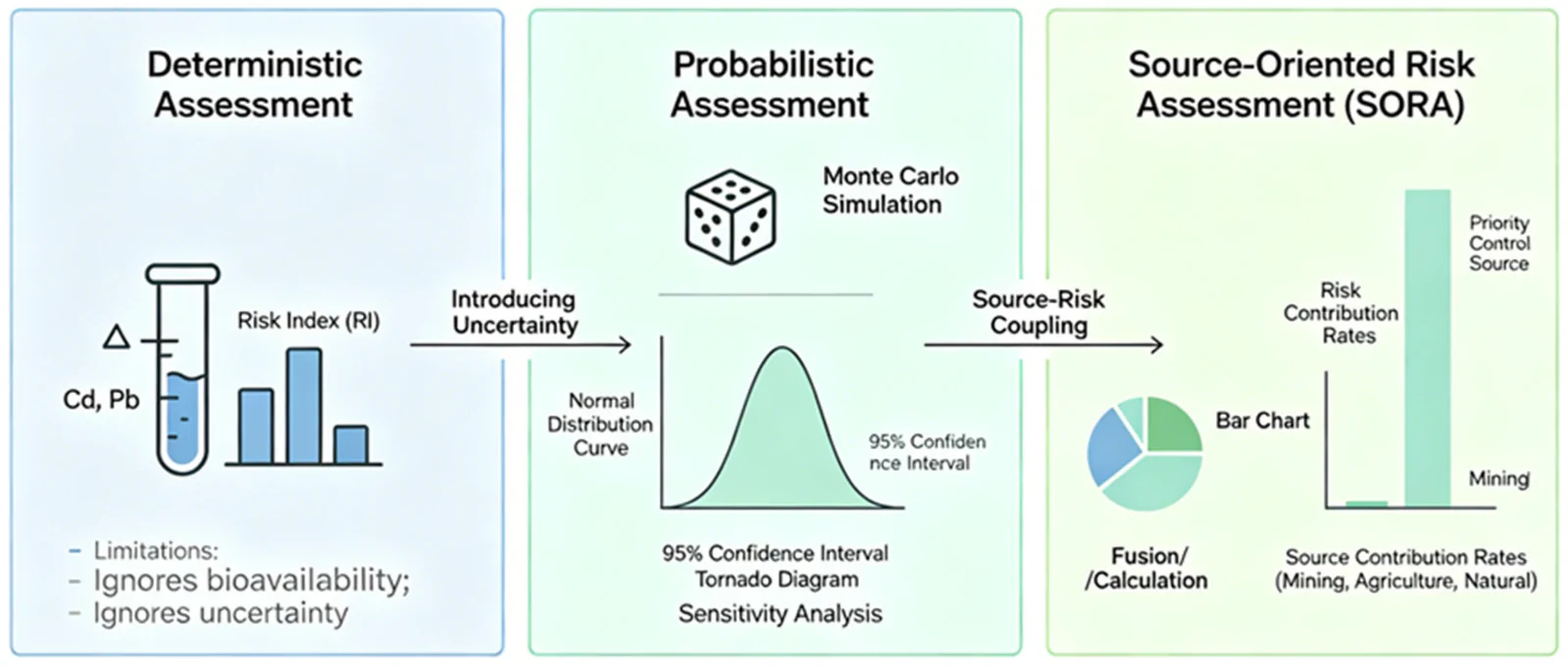
Conceptual framework of risk assessment evolution for heavy metals in mining area soils. Schematic illustration showing the progressive risk assessment framework exemplified by Cd and Pb. This figure illustrates the progression from traditional deterministic risk assessment (using fixed threshold values) to modern probabilistic risk assessment (incorporating Monte Carlo simulation), and further to source-oriented risk assessment that identifies priority control sources.

The ultimate harm of heavy metal pollution in mining area soils lies in its threat to ecosystem structure and human health. Traditional risk assessment is mostly based on deterministic single-point value models. Although simple to calculate, when facing the extremely high spatial heterogeneity of mining area soils and the uncertainty of exposure parameters, it often produces overly conservative or optimistic results, making it difficult to effectively support scientific decision-making. With the development of models, risk assessment is transforming from deterministic description to probabilistic characterization and further integrating deeply with source apportionment, forming a comprehensive risk management system of “source–pathway–receptor” (Figure 4).

### 5.1. Deterministic Risk Assessment System and Its Limitations

Currently, the risk assessment of HMs in mine soils mainly adopts pollution index methods (geo-accumulation index, Nemerow comprehensive index), potential ecological risk index (RI) and USEPA health risk assessment model. These deterministic approaches have been extensively applied in diverse contexts [100]. In ultramafic rock areas, the geo-accumulation index and Hakanson RI were utilized to evaluate pollution levels and ecological risks associated with Co, Cr, and Ni, highlighting the mobility and bioavailability of these metals in soil systems [101]. In China’s main grain-producing regions, deterministic health risk assessments have revealed that heavy metal(loid)s in agricultural soils pose potential risks to human populations, with bioaccessibility data providing critical supplementary information for more accurate risk characterization [102]. However, these deterministic methods have inherent limitations: they rely on fixed threshold values that do not account for spatial variability, and they cannot quantify uncertainty in exposure parameters.

### 5.2. Uncertainty Analysis and Probabilistic Risk Characterization

To address the inherent limitations of traditional risk assessment models for HMs in mine soils, the introduction of uncertainty analysis has become a consensus in this field. Monte Carlo simulation, for instance, has been widely applied to propagate parameter uncertainties through risk models. Generally, soil heavy metal concentrations follow log-normal distribution, while exposure parameters follow triangular or uniform distribution. Monte Carlo simulation should be run for no less than 10,000 iterations to ensure result stability [103,104]. In China’s main grain production regions, probabilistic risk assessment incorporating bioaccessibility measurements has been applied to evaluate heavy metal(loid) contamination in agricultural soils, revealing that bioaccessibility-based risk estimates provide a more realistic characterization of human health risks compared to total content-based deterministic approaches [105]. The Monte Carlo simulation-triangular fuzzy number coupling model has been devel-oped to assess heavy metal risks in agricultural soils, demonstrating that the probabil-istic approach provides a more realistic risk range (95% confidence interval) compared to deterministic single-point estimates [106]. 

### 5.3. Source-Risk Coupling: From “Pollution Source Tracing” to “Risk Source Tracing”

Traditional source apportionment can only identify the origin of pollutants but fails to address the core concern of environmental managers, i.e., which source contributes the most risk. Source-oriented risk assessment, which directly integrates the results of source apportionment models such as PMF into risk assessment models, represents a major recent breakthrough in the study of HMs in mine soils. Its core logic is to calculate the contribution percentage of each pollution source to the total potential ecological risk index (RI) or total non-carcinogenic hazard index (HI) based on the contribution rates and source profiles of each pollution source resolved by the PMF model, combined with the corresponding toxic effect coefficients [2,22]. Research on a gold smelting area indicated that tailings residue sources contributed more than 60% of the ecological risk for ecosystem protection, while traffic emission sources were the primary risk contributors for human health protection due to their high Pb content and easy enrichment in road dust. The establishment of this “source–risk” bidirectional transmission mechanism has directly informed strategies for identifying priority sources and priority pollutants. Based on this strategy, managers no longer blindly remediate the entire area but can concentrate funds and technologies for precise targeting. This finding directly overturns the intuitive judgment that “higher content means higher risk”, highlighting the scientificity and economic efficiency of control based on risk contribution [52]. 

## 6. Research Progress on Sustainable Remediation Technologies

Traditional heavy metal remediation technologies for mining area soils (such as soil replacement method and chemical washing) often face problems such as high energy consumption, high secondary pollution risk, or destruction of soil structure. With the rise of the “Green Remediation” concept, researchers have begun to pay more attention to the environmental friendliness, cost-effectiveness, and long-term ecological stability of remediation technologies [107,108]. This section systematically reviews microbial remediation, phytoremediation, and their cross-integration with materials science, exploring their application potential and challenges in achieving sustainable management of mining area soils.

### 6.1. Microbial Remediation Technology

Microbial remediation employs the metabolic activities of specific microorganisms (such as bacteria and fungi) in soil to convert heavy metal ions into low-toxic or non-toxic forms through biosorption, redox reactions, biomineralization, etc., thereby reducing their mobility and bioavailability in soil [109].

**Figure 5:**
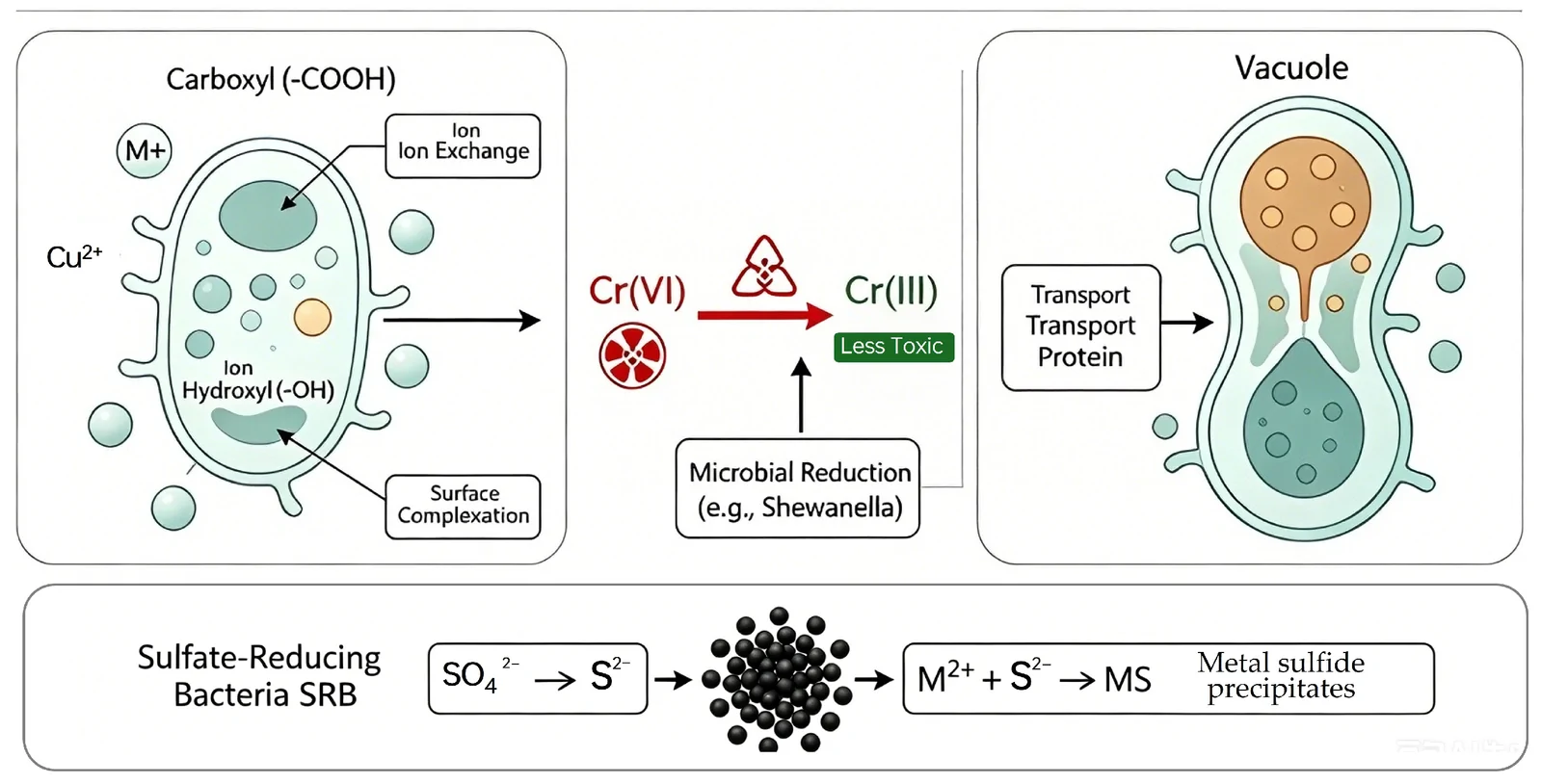
Mechanism of microbial remediation of Cd, Pb, and As in mining area soils. This figure illustrates the key mechanisms of microbial remediation: (1) biosorption by PGPR bacteria and SRB; (2) redox reactions converting toxic Cr (VI) to less toxic Cr(III); (3) precipitation forming stable metal sulfides; and (4) bioaccumulation within microbial cells. The mechanisms are particularly effective for Cd and Pb immobilization in acidic mining soils, with SRB showing promising results in AMD-impacted areas.

The core of microbial remediation lies in using its metabolic activity or cellular characteristics to change the environmental behavior of HMs. The main mechanisms include biosorption, redox reactions, and biomineralization (Figure 5) [110]. Studies show that functional groups (such as carboxyl, hydroxyl, and amino groups) on the surface of microbial cells rapidly complex heavy metal ions through biosorption [111]. This process does not depend on metabolic activity, and dead bacteria are also effective. For example, selected *Bacillus subtilis* and *Pseudomonas aeruginosa* can adsorb free Cd within hours, creating conditions for subsequent plant colonization [112]. In terms of redox reactions, *Shewanella* and *Geobacter* can reduce highly toxic Cr (VI) to slightly toxic Cr(III); Fe(III) oxides produced by iron-oxidizing bacteria can indirectly immobilize HMs such as Pb and As through co-precipitation [113,114,115]. In contrast, biomineralization represents a more thorough in situ solidification strategy. Under anaerobic conditions, sulfate-reducing bacteria (SRB) reduce SO_4_^2−^ to S^2−^, which combines with Pb^2+^, Cd^2+^, Zn^2+^, etc. to form extremely insoluble metal sulfide precipitates (Figure 5). Due to the extremely high thermodynamic stability of sulfide minerals in the environment, this method can not only reduce bioavailability but also achieve long-term risk management, and it is considered one of the most promising biotechnologies for treating acidic sulfate soils in mining areas [116,117].

### 6.2. Phytoremediation Technology

#### 6.2.1. Hyperaccumulator Plants and Their Applications

Phytoremediation is a green technology that uses hyperaccumulators’ extraordinary ability to absorb, transport, and accumulate HMs from soil to remove pollutants [118]. Hyperaccumulator plants are plants that can accumulate abnormally high concentrations of HMs in their aboveground parts (stems, leaves) without showing obvious toxic symptoms. This characteristic makes them star species for mining area soil remediation. In Pb-Zn mining areas in southern China, due to the widespread combined pollution of Cd and Zn in soils, *Sedum alfredii* and *Sedum plumbizincicola* have become the first choice for research and application due to their strong extraction ability for these two metals [119,120,121]. In contrast, in mining areas heavily polluted with As such as Hunan and Guangxi, *Pteris vittata* shows unique advantages, with As content in its aboveground parts exceeding 1000 mg/kg, and it can transfer pollutants from roots to stems and leaves through efficient transport mechanisms [122,123]. The advantages of phytoremediation include low cost, minimal soil disturbance, and landscape beautification functions. However, its limitations are also obvious; a long remediation cycle applies (usually requiring several years or even decades), and most hyperaccumulator plants have small biomass and slow growth, making them suitable only for moderately to lightly polluted soils [124]. For heavily polluted mining areas, single phytoremediation extraction often cannot meet the remediation time requirements.

#### 6.2.2. Phytoextraction and Phytostabilization

**Figure 6:**
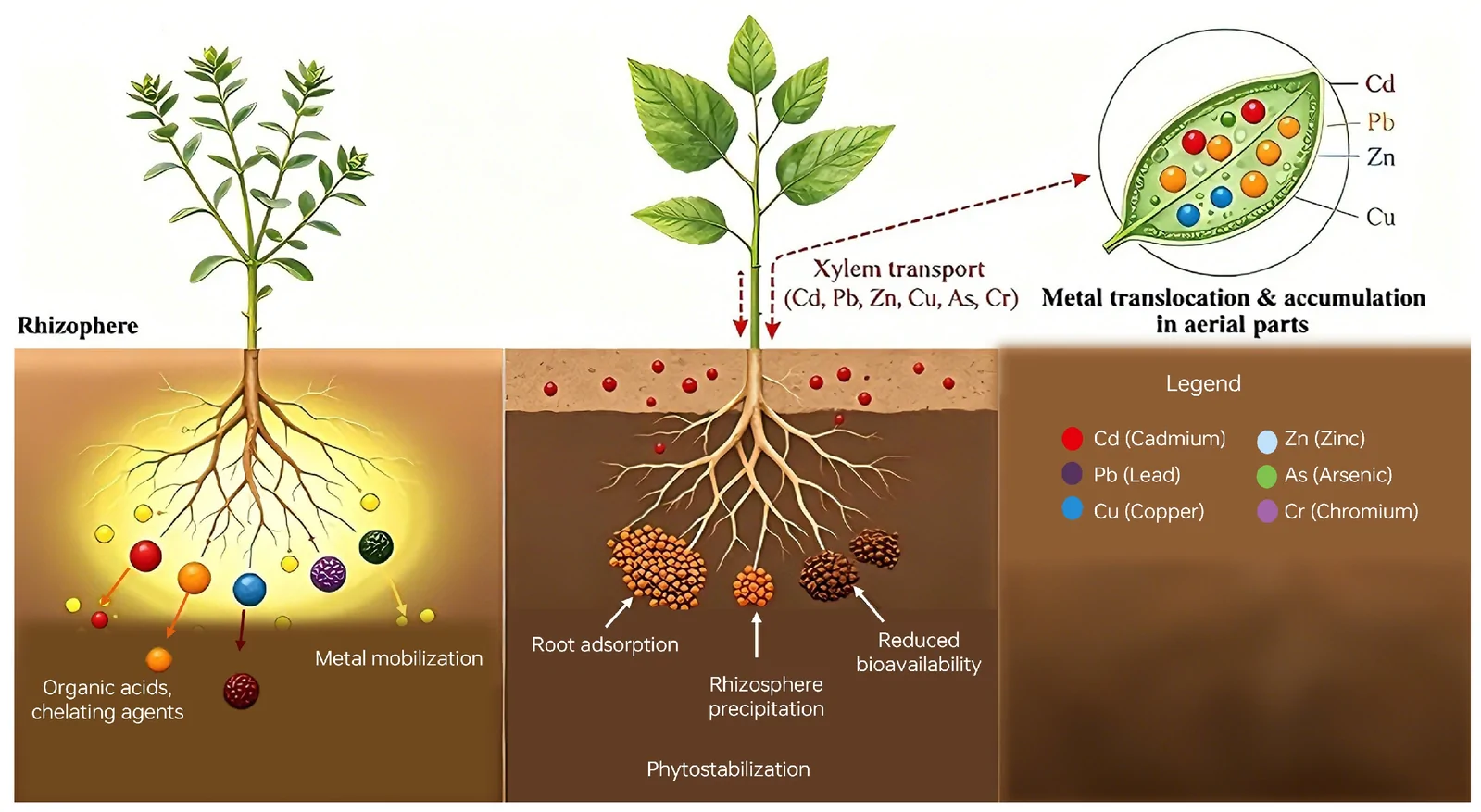
Schematic diagram of phytoremediation mechanisms (phytoextraction vs. phytostabilization) for Cd, Pb, and Zn in mining area soils. This figure illustrates the two primary phytoremediation strategies: (1) phytoextraction using hyperaccumulator plants (*Sedum alfredii*, *Sedum plumbizincicola* for Cd/Zn; *Pteris vittata* for As) that absorb and accumulate heavy metals in aboveground biomass for subsequent harvest and removal; and (2) phytostabilization using plants (*Athyrium wardii*) that immobilize heavy metals in rhizosphere soil through root adsorption, precipitation, or oxidation, reducing bioavailability and migration risk.

According to different mechanisms, phytoremediation is mainly divided into phytoextraction and phytostabilization (Figure 6) [125,126]. The goal of phytoextraction is to transfer HMs from soil to plant aboveground parts and then harvest plants for incineration or landfill to achieve permanent removal of pollutants. This method is suitable for moderately to lightly polluted areas with high pollutant activity. However, for high-concentration, high-risk contaminated sites, simple removal may face low efficiency or risk of secondary diffusion. In this case, phytostabilization technology is more valuable [107,127,128,129,130]. It uses plant root adsorption, precipitation, or oxidation to immobilize HMs in rhizosphere soil, reducing their bioavailability and migration risk to groundwater. Through the physical consolidation of roots and chemical regulation of the rhizosphere microenvironment, phytostabilization can effectively prevent the spread of pollution plumes, gaining time for subsequent in-depth treatment or risk management [131,132].

### 6.3. Material-Assisted Chemical-Biological Combined Remediation

#### 6.3.1. Biochar-Assisted Synergistic Remediation

**Figure 7:**
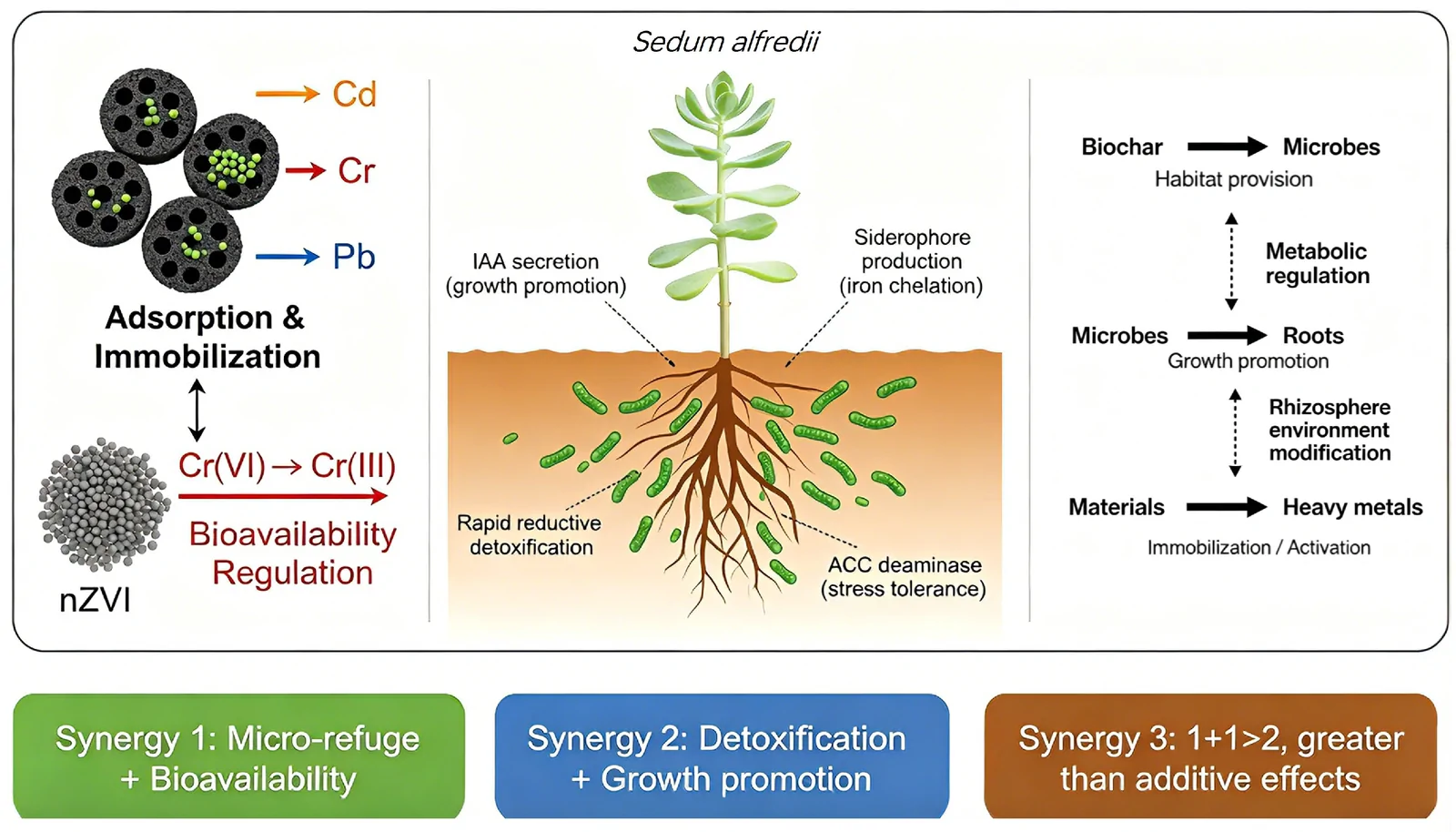
Schematic diagram of biochar–nanomaterial-assisted phytoremediation mechanisms for Cd, Pb and Cr in mining area soils. This figure demonstrates the synergistic remediation effects of composite amendments and *Sedum alfredii*. (1) Biochar adsorbs and immobilizes Cd and Pb, and nano-zero-valent iron (nZVI) reduces Cr(VI) to Cr(III) to regulate heavy metal bioavailability; (2) Biochar-colonized rhizosphere microorganisms secrete IAA, siderophores and ACC deaminase to facilitate detoxification and relieve heavy metal stress of *Sedum alfredii*; (3) Interactions among biochar, microbes, plant roots and functional materials modulate the rhizosphere environment for combined heavy metal immobilization.

Single microbial remediation or phytoremediation often faces bottlenecks due to its long cycle and low efficiency. Introducing environmental functional materials (such as biochar and nanomaterials) into the remediation system to form a “material–plant–microbe” multi-phase synergistic system is a hot topic in current research into mining area soil remediation (Figure 7) [133,134,135].

Biochar has strong adsorption and immobilization capabilities for various HMs such as Cd, Pb, and Cu due to its huge specific surface area, rich pore structure, and surface functional groups (such as carboxyl and phenolic hydroxyl groups). In acidic mining area soils, the “liming effect” of biochar can significantly increase soil pH, promoting the formation of heavy metal hydroxide precipitates. In addition, the rich soluble organic matter in biochar can form complexes with HMs, further reducing their bioavailability [136,137]. However, biochar’s role in the combined remediation system is far more than just a simple adsorbent; it is also a “micro-refuge” for microbial habitat. Facing the challenge of biological survival under high pollution concentrations, biochar quickly adsorbs and immobilizes free heavy metal ions in soil, reducing the toxicity of the rhizosphere environment and providing a buffered “safe island” for sensitive microorganisms and plant seedlings, thereby achieving the goal of detoxification and efficiency enhancement [137]. At the same time, the porous structure of biochar can protect microorganisms from predation by soil protozoa and provide attachment surfaces for them, significantly improving the survival rate and action time of functional microbial agents (such as PGPR and SRB) [138]. In addition, biochar can also create a more favorable microenvironment for plant root growth and microbial activity by adjusting soil C/N ratio, retaining moisture, and promoting aggregate formation [139]. Two-year field trials on Cd-contaminated paddy soils have verified that rice straw biochar remarkably decreases soil available Cd and Cd accumulation in rice grains, with biochar amendments reducing Cd concentrations in rice grains and soil available Cd by 16.8–61.9% and 32.0–52.5%, respectively [140,141]. Further results illustrated that carbon-based modified materials reduce DTPA-extractable Cd by 79.69%, effectively inhibiting Cd translocation into rice grains [142]. These field studies confirm the remarkable efficacy of biochar in remediating heavy metal pollution in mining-impacted agricultural soils.

#### 6.3.2. Nanomaterial-Assisted Enhanced Remediation

Nanomaterials (such as nano-zero-valent iron, nano-hydroxyapatite) show great potential for heavy metal remediation due to their size effect and extremely high reactivity (Figure 7). Among them, nano-zero-valent iron (nZVI) has strong reducing ability, which can reduce Cr(VI) to Cr(III), and immobilize them through surface adsorption and co-precipitation, making it particularly suitable for treating chromium slag sites and arsenic-contaminated soils [143,144,145]. Nano-hydroxyapatite (nHAP) has good immobilization effects on Pb and Cd through surface adsorption and ion exchange, and has high biocompatibility. In combined remediation, nanomaterials mainly play roles in “pretreatment” and “speciation regulation”. Applying nanomaterials in the early stage of remediation can quickly reduce the concentration of HMs in soil solution, alleviate acute toxicity, and gain time for subsequent plant colonization and microbial community establishment [146,147]. More ingeniously, some nanomaterials (such as modified nano-Fe_3_O_4_) can directionally change the speciation of HMs. For example, they can transform residual or bound HMs that are difficult for plants to utilize into plant-available forms (activation) through surface catalysis or redox reactions, thereby improving phytoextraction efficiency; or conversely, transform active HMs into stable forms (passivation) for phytostabilization [148,149]. In addition, nanoparticles are also explored as carriers for microorganisms or enzymes to achieve targeted delivery and protection.

### 6.4. Sustainability Assessment of Remediation Technologies

Faced with numerous remediation technology options, how to conduct scientific screening and decision-making is a key issue in environmental management. Sustainability Assessment no longer only focuses on heavy metal removal efficiency, but introduces the “triple bottom line” principle, which conducts comprehensive evaluation according to three dimensions: environmental benefits, economic benefits, and social benefits [150,151,152].

The implementation of remediation technologies relies on community support, and public perception and acceptance of technologies directly affect project progress. The public has higher trust in “visible” physical engineering and tends to have doubts about green technologies with long cycles and gradual effects; for farmland or residential sites, the use of chemical agents often causes public sensitivity, making green remediation technologies more advantageous in the social dimension [151]. Economic cost is the main bottleneck restricting the large-scale application of remediation technologies. Phytoremediation has low initial investment but a long cycle, and field management costs cannot be ignored; chemical immobilization materials have higher costs but quick effects, which can reduce subsequent monitoring costs [152]. More and more studies have focused on the economic value-added potential of nature-based solutions. Planting energy-type hyperaccumulator plants can achieve “phytomining”, and their biomass can produce biochar or biomass energy. In the future, carbon sink value in the carbon trading market may offset part of the remediation costs [152]. Although green remediation is a future trend, in situ chemical passivation (phosphate rock powder, lime, sepiolite, etc.) is still the most widely used method in large-scale mining area remediation in China. Its costs are controllable, cycle is short, and it can quickly block food chain exposure risks. In practical engineering decisions, a phased strategy is often adopted, initially using chemical passivation for rapid hemostasis, and after risks are reduced, introducing green technologies such as plant-microbe remediation for long-term ecological restoration.

In summary, the future development direction of heavy metal remediation in mining area soils is to build a multi-technology integration system centered on green sustainability, through the organic combination of biochar improvement, functional microbial agent enhancement, and hyperaccumulator plant extraction, achieving the transformation from “end-of-pipe treatment” to ecological reconstruction.

## 7. Discussion and Limitations

### 7.1. Discussion

This section discusses three key aspects. (1) Regarding the causes of regional differences in pollution characteristics, the spatial heterogeneity of heavy metal pollution in Chinese mining areas is shaped by the combined effects of geological background, mining type, and anthropogenic activities. Southwest karst areas face unique challenges due to high natural background values and fragile ecosystems. (2) With reference to sources of uncertainty in source apportionment and risk assessment, different receptor models have varying performance in high-background areas, and uncertain exposure parameters have a significant effect on risk characterization. (3) Considering the barriers to and strategies for sustainable remediation, high cost, long remediation cycles, and public acceptance are major barriers to implementing sustainable remediation technologies in Chinese mining areas. Additionally, we acknowledge the heterogeneity in research findings, particularly regarding biochar effects on metal bioavailability, which may be attributed to biochar properties, soil types, and metal speciation.

As a complex system issue involving multiple disciplines, research into heavy metal pollution in mining area soils currently still faces challenges such as difficult precise remediation due to site spatial heterogeneity, lack of differentiated assessment systems for high-background-value areas, insufficient data on combined pollution interaction effects and long-term remediation stability, and imperfect sustainable remediation evaluation index systems. In the future, it will be necessary to transform towards “smartization”, to achieve dynamic early warnings and coupled simulation through high-density in situ sensors, satellite remote sensing, and artificial intelligence, and to develop synergistic remediation systems with multi-technology coupling such as “electro-microbial remediation” and “chemical washing–phytoremediation”. At the policy level, it is necessary to establish a differentiated soil environmental quality standards system (adopting a “baseline value + increment” assessment model for high-background-value areas), implement full-chain “pollution source–risk–remediation” management, improve policy support for sustainable remediation technologies (by establishing special funds, establishing certification catalogs, and exploring “remediation + carbon sink” business models), strengthen monitoring and early warning capabilities, and enhance cross-departmental coordination and regional linkage. These measures can facilitate the sustainable utilization of soil resources and promote the green ecological development of mining areas.

### 7.2. Limitations

This review has several limitations that should be acknowledged. First, there is a language bias in the included studies, with the majority of references being published in English or Chinese, potentially overlooking relevant research in other languages. Second, publication bias may exist, as studies with significant or positive findings are more likely to be published. Third, the reviewed studies exhibit methodological variations in sampling strategies, analytical methods, and data reporting standards, which may affect the comparability of results. Fourth, this study mainly focuses on Cd, Pb, As, and Hg, and does not cover emerging contaminants such as Tl and Sb. Regarding the limitations of studies into Chinese mining and soil, many existing investigations focus on single mining sites or specific regions, lacking national-scale comprehensive assessments. Additionally, data availability remains a challenge, particularly for historical mining areas and remote regions. Methodological constraints include inconsistent pollution assessment standards and limited long-term monitoring data, which hinder accurate trend analysis and predictive modeling.

### 7.3. Policy Implications

Based on the findings of this review, we propose the following policy implications: (1) Establish differentiated soil environmental quality standards for southwestern karst areas, conducting risk assessment based on regional geochemical baselines; (2) Implement a “source–risk–remediation” whole-chain management model, requiring source-oriented risk assessment for all mining development projects; (3) Set up special funds for sustainable remediation technology research and industrialization, supporting applications of biochar, PGPR agents, and other green technologies; (4) Strengthen monitoring networks in mining areas, establishing long-term soil quality monitoring systems; (5) Promote cross-sectoral collaboration among environmental protection, land resources, and mining industries to develop integrated management policies.

## 8. Conclusions

This study systematically demonstrates that soil heavy metal pollution characteristics in China’s metal mining areas are fundamentally shaped by the combined effects of inherent geological backgrounds and anthropogenic disturbances, with regional variations driven by metallogenic conditions, mining technologies, and environmental factors. However, current source apportionment and risk assessment studies are plagued by significant uncertainties and heterogeneity of results due to methodological limitations, parameter differences, and environmental variability. Furthermore, the practical application of sustainable remediation technologies faces multiple technical, economic, policy, and social barriers that hinder large-scale implementation. Future research should prioritize the development of intelligent multi-source pollution tracing and dynamic risk early warning systems for high geological background areas, and an integrated ecosystem restoration and value-added framework should be established aligned with the “mountains, waters, forests, farmlands, lakes, and grasslands” life community concept. This review provides a comprehensive synthesis of current research progress and proposes targeted strategies for uncertainty control and integrated remediation, offering a scientific basis for the prevention and control of heavy metal pollution in mining areas.

## Figures and Tables

**Table 1:** Comparison of mainstream source apportionment methods for HMs in mining area soils.

Method Type	Specific Method	Basic Principle	Advantages	Limitations	References
Multivariate Statistics	PCA/FA	Principal component analysis extracts potential source factors	Simple calculation, easy to interpret results	Cannot quantify source contributions, prone to misjudgment under high background values	[93]
Multivariate Statistics	APCS-MLR	Absolute principal component scores combined with multiple regression	Can quantify source contributions, testable results	Assumes linear relationships, poor fitting for nonlinear systems	[94]
Receptor Models	PMF	Positive matrix factorization with non-negativity constraints	Provides uncertainty estimation, robust results	Subjective factor number selection, non-unique source profiles	[2,95]
Receptor Models	Unmix	Geometric method for identifying source profiles	No prior knowledge required	Sensitive to outliers, difficult to estimate source number	[95]
Machine Learning	SOM	Self-organizing map, unsupervised clustering	Visualizes spatial gradients, identifies pollution patterns	Sensitive to parameter selection, limited interpretability	[96]
Machine Learning	SOM-PMF	First cluster then regional PMF	Handles highly heterogeneous areas, improved accuracy	Complex calculation, requires large sample size	[97]
Machine Learning	Random Forest/Gradient Boosting Tree	Ensemble learning, importance ranking	Distinguish natural vs. anthropogenic sources, nonlinear fitting	Black-box model, unclear physical meaning	[81,98]
Isotope Technology	Pb/Sr Isotopes	Isotope fingerprint tracing	Directly distinguishes geological vs. anthropogenic sources	High analysis cost, limited sample size	[99]
Microscopic Characterization	TEM-EDS/XRD	Mineralogical microscopic evidence	Provides visual pollution evidence	Difficult to quantify, limited representativeness	[52]

## Data Availability

The original contributions presented in this study are included in the article/Supplementary Materials. Further inquiries can be directed to the corresponding author.

## Supplementary Materials

The following supporting information can be downloaded at https://www.mdpi.com/article/10.3390/toxics14080689/s1, Figure S1: PRISMA 2020 Flowchart of Literature Search and Screening Process; Table S1: Summary of All 152 Included Studies.

## References

[1] Xie S., Yu Y., Xie Y., Chen X., Tang Z. (2025). Overview and prospects of modeling and optimal control for non-ferrous metallurgical processes and mineral processing. Green Smart Min. Eng..

[2] Zhou Y., Ding D., Zhao Y., Li Q., Jiang D., Lv Z., Wei J., Zhang S., Deng S. (2024). Determining priority control toxic metal for different protection targets based on source-oriented ecological and human health risk assessment around gold smelting area. J. Hazard. Mater..

[3] Ha N.T.H., Hai N.T., Nhung T.K., Anh B.T.K., Nga T.T.H., Kim K.W. (2026). A review and meta-analysis on heavy metals in soils and crops at metal mines: Accumulation, source, food safety, health risk, and management. J. Hazard. Mater. Adv..

[4] Shi J., Liu J., Hu S., Wang J. (2025). Distribution Characteristics of High-Background Elements and Assessment of Ecological Element Activity in Typical Profiles of Ultramafic Rock Area. Toxics.

[5] Zhao X., Yang Z., Yu F. (2023). Review on heavy metal pollution and remediation technology in the soil of mining. Geol. China.

[6] Yang W., Zhang L., Gao B., Liu X., Duan X., Wang C., Zhang Y., Li Q., Wang L. (2024). Integrated assessment of potentially toxic elements in soil of the Kangdian metallogenic province: A two-point machine learning approach. Ecotoxicol. Environ. Saf..

[7] Xu D.M., Fu R.B., Liu H.Q., Guo X.P. (2021). Current knowledge from heavy metal pollution in Chinese smelter contaminated soils, health risk implications and associated remediation progress in recent decades: A critical review. J. Clean. Prod..

[8] Wei N., Gu X., Wen Y., Guo C., Ji J. (2025). Geochemical speciation and activation risks of Cd, Ni, and Zn in soils with naturally high background in karst regions of southwestern China. J. Hazard. Mater..

[9] Xu S., Yun M., Wang Y., Liu K., Wu A., Li S., Su Y., Wang S., Kang H. (2025). Heavy Metal Contamination and Risk Assessment in Soil-Wheat/Corn Systems near Metal Mining Areas in Northwestern China. Biology.

[10] Yan Y., Mao Z., Wang X., Chen Z., Ma C., Wei D., Yan W., Wu X., Guo Y., Xu H. (2025). From farm to table: Assessing the status and health risk assessment of heavy metal pollution in rice in Henan Province. Front. Public Health.

[11] Deng M., Zhu Y., Shao K., Zhang Q., Ye G., Shen J. (2020). Metals source apportionment in farmland soil and the prediction of metal transfer in the soil-rice-human chain. J. Environ. Manag..

[12] Landrigan P.J., Baker E.L. (1981). Exposure of children in heavy metals from smelters: Epidemiology and toxic consequences. Environ. Res..

[13] Sun Y., Yang Z., Dong K., Hui F., Wang D., Huang Y. (2025). Pollution Risk Assessment of Potentially Toxic Elements in Soils Using Characterization and Microbiological Analysis: The Case of a Rare and Precious Metal Mining Site in Wuzhou, Guangxi. Toxics.

[14] Song L., Zhang T., Yan H., Xu J., Chen W., Ba Y., Wang H., Qian K., Li Y., Wu W. (2026). Heavy Metals Risk Assessment and Source Apportionment in Agricultural Soils of the Central Yunnan Dry-Hot Valley. Toxics.

[15] Xu Y., Wang X., Cui G., Li K., Liu Y., Li B., Yao Z. (2022). Source apportionment and ecological and health risk mapping of soil heavy metals based on PMF, SOM, and GIS methods in Hulan River Watershed, Northeastern China. Environ. Monit. Assess..

[16] Lv X., Li C., Wang H., Yang S., Wang S., Yang C., Teng F., Zhao J., Xu X., Zhang H. (2025). Source apportionment and risk quantification of soil heavy metals using SOM-PMF model: Implications for ecological and human health management. Envron. Monit. Assess..

[17] Lin Y., Liu Y., Tan Y., Wu N., Wang L., Chen J., Sun X., Dong X., Chen B., Pan Z. (2026). Source apportionment and ecological risks of trace metals in multiple media in a typical Chinese industrialized bay on the basis of SOM, PMF and GIS methods. Mar. Pollut. Bull..

[18] Wu Y., Xia Y., Mu L., Liu W., Wang Q., Su T., Yang Q., Milinga A., Zhang Y. (2024). Health Risk Assessment of Heavy Metals in Agricultural Soils Based on Multi-Receptor Modeling Combined with Monte Carlo Simulation. Toxics.

[19] Ma J., Lanwang K., Liao S., Zhong B., Chen Z., Ye Z., Liu D. (2023). Source Apportionment and Model Applicability of Heavy Metal Pollution in Farmland Soil Based on Three Receptor Models. Toxics.

[20] Yuan B., Cao H., Du P., Ren J., Chen J., Zhang H., Zhang Y., Luo H. (2023). Source-oriented probabilistic health risk assessment of soil potentially toxic elements in a typical mining city. J. Hazard. Mater..

[21] Xie G., Zong Z., Song S., Lei X., Liu W., Song C., Xie G. (2025). Enrichment and Source Apportionment of Heavy Metals in Selenium-Enriched Soils in Eastern China. ACS Omega.

[22] Huang W., Liu Y., Bi X., Wang Y., Li H., Qin J., Chen J., Ruan Z., Chen G., Qiu R. (2025). Source-specific soil heavy metal risk assessment in arsenic waste mine site of Yunnan: Integrating environmental and biological factors. J. Hazard. Mater..

[23] Ni M., Yan S., Li T., Zhou Z., Liu X., Zhang S., Wang G., Xu X., Pu Y., Jia Y. (2025). Identifying priority control factors for heavy metal pollution in urban agricultural soils of the Chengdu Plain: An integrated multi-model approach. Ecotoxicol. Environ. Saf..

[24] Sen T.K. (2023). Agricultural Solid Wastes Based Adsorbent Materials in the Remediation of Heavy Metal Ions from Water and Wastewater by Adsorption: A Review. Molecules.

[25] Wang L., Rinklebe J., Tack F.M., Hou D. (2021). A review of green remediation strategies for heavy metal contaminated soil. Soil Use Manag..

[26] Yang S., Ning Y., Li H., Zhu Y. (2024). Effects of *Priestia aryabhattai* on Phosphorus Fraction and Implications for Ecoremediating Cd-Contaminated Farmland with Plant-Microbe Technology. Pharmaceuticals.

[27] Raklami A., Meddich A., Oufdou K., Baslam M. (2022). Plants-Microorganisms-Based Bioremediation for Heavy Metal Cleanup: Recent Developments, Phytoremediation Techniques, Regulation Mechanisms, and Molecular Responses. Int. J. Mol. Sci..

[28] Saxena G., Purchase D., Mulla S.I., Saratale G.D., Bharagava R.N. (2020). Phytoremediation of Heavy Metal-Contaminated Sites: Eco-environmental Concerns, Field Studies, Sustainability Issues, and Future Prospects. Rev. Environ. Contam. Toxicol..

[29] Zheng K., Liu Z., Liu C., Liu J., Zhuang J. (2023). Enhancing remediation potential of heavy metal contaminated soils through synergistic application of microbial inoculants and legumes. Front. Microbiol..

[30] Krishna Raj C., Siranjeevi R., Susmitha R., Sameera Shabnum S., Saravanan A., Vickram A.S., Chopra H. (2025). Bioremediation in action: Recent progress, challenges, and future directions for environmental restoration. Biodegradation.

[31] Firincă C., Zamfir L.-G., Constantin M., Răut I., Jecu M.-L., Doni M., Gurban A.-M., Șesan T.E. (2025). Innovative Approaches and Evolving Strategies in Heavy Metal Bioremediation: Current Limitations and Future Opportunities. J. Xenobiot..

[32] Jha A., Barsola B., Pathania D., Sonu, Raizada P., Thakur P., Singh P., Rustagi S., Khosla A., Chaudhary V. (2024). Nano-biogenic heavy metals adsorptive remediation for enhanced soil health and sustainable agricultural production. Environ. Res..

[33] Wang X., Deng R., Wang C., Xu R., Hou B., Zhou S., Wang X., Ren B., Hursthouse A. (2025). Pollution characteristics, risk assessment and source comparative analysis of potentially toxic elements in soils of three superlarge antimony ore areas in the Zijiang River, Hunan, China. Environ. Pollut..

[34] García G., Rosique G. (2025). Underlying Causes of Long-Term Environmental Pollution by Waste from an Abandoned Metal Mining District: When Legislative and Remediation Measures Are Ineffective. Environments.

[35] Xu M., Lei M., Ju T., Guo G., Xie Y., Shi P. (2026). Causal inference-driven framework for source verification: Integrating PMF, health risk assessment, and spatialized emission inventories, and in multi-source composite heavy metal soil pollution. J. Hazard. Mater..

[36] Li P., Lin C., Cheng H., Duan X., Lei K. (2015). Contamination and health risks of soil heavy metals around a lead/zinc smelter in southwestern China. Ecotoxicol. Environ. Saf..

[37] Yang L., Bai Z., Bo W., Lin J., Yang J., Chen T. (2024). Analysis and Evaluation of Heavy Metal Pollution in Farmland Soil in China:A Meta-analysis. Huan Jing Ke Xue.

[38] Meng X.F., Guo J.M., Yang J.X., Yang J., Zheng G.D., Qao P.W., Bian J.L. (2021). Spatial Distribution and Risk Assessment of Heavy Metal Pollution in Farmland Soils Surrounding a Typical Industrial Area of Henan Province. Huan Jing Ke Xue.

[39] Xia B., Gou W., Huang Y., Chen Y., Zhu Z., Pei X., He M., Duo J. (2025). Identifying hidden heavy metal sources in atmospheric dust of mining cities by integrating Cd isotopes and multivariate statistical method. J. Hazard. Mater..

[40] Zhang J.L., Qu M.K., Chen J., Yang L.F., Zhao Y.C., Huang B. (2021). Meta-analysis of the Effects of Metal Mining on Soil Heavy Metal Concentrations in Southwest China. Huan Jing Ke Xue.

[41] Zhu D., Li Q., Yan B. (2026). Assessment of Heavy Metal Contamination Levels and Health Risks in a Mining Area in Sichuan Province, China. Pol. J. Envron. Stud..

[42] Hui J., Cheng Y., Zhou Y., Li S. (2026). Integrating soil heavy metal risk into ecological quality assessment: The pollution-enhanced remote sensing ecological index. J. Hazard. Mater..

[43] He J., Peng Z.H., Zeng J.Q., Li C.X., Tang L., Jiang J., Luo X.H., Gao W.Y., Guo J.K., Shao B.B. (2023). Source apportionment and quantitative risk assessment of heavy metals at an abandoned zinc smelting site based on GIS and PMF models. J. Environ. Manag..

[44] Chen R., Han L., Liu Z., Zhao Y., Li R., Xia L., Fan Y. (2022). Assessment of Soil-Heavy Metal Pollution and the Health Risks in a Mining Area from Southern Shaanxi Province, China. Toxics.

[45] Jiang Y., Ma J., Wang Y., Yang Y. (2025). Background Values of Soil Heavy Metals in the Huang-Huai-Hai Plain in Henan Province, China. Toxics.

[46] Li Q., Han Z., Tian Y., Xiao H., Yang M. (2023). Risk Assessment of Heavy Metal in Farmlands and Crops Near Pb-Zn Mine Tailing Ponds in Niujiaotang, China. Toxics.

[47] Hu F., Wang B., Zhang L., Wang Y., Sha J., Dong J., Song H., An J. (2026). Ecotoxicological Effects of Heavy Metals on Rice (*Oryza sativa* L.) Across Its Life Cycle and Health Risk Assessment in Soils Around Pb–Zn Mine. Plants.

[48] Zang Z., Li Y., Li H., Guo Z., Zhang R. (2020). Spatiotemporal Variation and Pollution Assessment of Pb/Zn from Smelting Activities in China. Int. J. Environ. Res. Public Health.

[49] Hua L., Yang X., Liu Y., Tan X., Yang Y. (2018). Spatial Distributions, Pollution Assessment, and Qualified Source Apportionment of Soil Heavy Metals in a Typical Mineral Mining City in China. Sustainability.

[50] Jin L., Ni Y., Liu X., Li Z., Xu X., Li Q., Yang Z., Luo L., Xie X. (2026). Sources, flux, and potential risks of heavy metals in soils around the smelting and mining area. J. Environ. Sci..

[51] Van Alphen M. (1999). Atmospheric heavy metal deposition plumes adjacent to a primary lead-zinc smelter. Sci. Total Environ..

[52] Guo G., Chen S., Zhang J., Lei M. (2025). Prioritizing pollution sources and targeted potentially toxic elements in soils using a probabilistic source-specific eco-health risk assessment from micro and macro perspectives. J. Hazard. Mater..

[53] Hou Y., Zhao Y., Lu J., Wei Q., Zang L., Zhao X. (2023). Environmental contamination and health risk assessment of potentially toxic trace metal elements in soils near gold mines-A global meta-analysis. Environ. Pollut..

[54] González-Valoys A.C., Arrocha J., Monteza-Destro T., Vargas-Lombardo M., Esbrí J.M., Garcia-Ordiales E., Jiménez-Ballesta R., García-Navarro F.J., Higueras P. (2022). Environmental challenges related to cyanidation in Central American gold mining; the Remance mine (Panama). J. Environ. Manag..

[55] Chen M.H., Yu X.Z., Feng Y.X. (2022). Tracing the pollution and human risks of potentially toxic elements in agricultural area nearby the cyanide baths from an active private gold mine in Hainan Province, China. Environ. Geochem. Health.

[56] Bai J., Xu X., Duan Y., Zhang G., Wang Z., Wang L., Zheng C. (2022). Evaluation of resource and environmental carrying capacity in rare earth mining areas in China. Sci. Rep..

[57] Wang L., Liang T. (2016). Anomalous abundance and redistribution patterns of rare earth elements in soils of a mining area in Inner Mongolia, China. Environ. Sci. Pollut. Res. Int..

[58] Guo Z., Liu Q., Luo F., Xie S., Zhou T. (2025). Study on the release pattern of Zn in soil of ionic rare earth mining areas under different leaching conditions. PLoS ONE.

[59] Götzke L., Schaper G., März J., Kaden P., Huittinen N., Stumpf T., Weigand J.J. (2019). Coordination chemistry of f-block metal ions with ligands bearing bio-relevant functional groups. Coord. Chem. Rev..

[60] Adnan M., Zhao P., Xiao B., Ali M.U., Xiao P. (2025). Heavy metal pollution and source analysis of soils around abandoned Pb/Zn smelting sites: Environmental risks and fractionation analysis. Environ. Technol. Innov..

[61] Chrastný V., Vaněk A., Teper L., Cabala J., Procházka J., Pechar L., Drahota P., Penížek V., Komárek M., Novák M. (2012). Geochemical position of Pb, Zn and Cd in soils near the Olkusz mine/smelter, South Poland: Effects of land use, type of contamination and distance from pollution source. Environ. Monit. Assess..

[62] Zhu Y., Li Q., Wu J., Chen X., Zhang J. (2025). Source-specific health risks of PM2. 5-bound heavy metals in a Chinese megacity impacted by non-ferrous metal mines. Atmos. Pollut. Res..

[63] Luo X., Wu C., Lin Y., Li W., Deng M., Tan J., Xue S. (2023). Soil heavy metal pollution from Pb/Zn smelting regions in China and the remediation potential of biomineralization. J. Environ. Sci..

[64] Zhou L., Wu F., Meng Y., Byrne P., Ghomshei M., Abbaspour K.C. (2023). Modeling transport and fate of heavy metals at the watershed scale: State-of-the-art and future directions. Sci. Total Envron..

[65] Thomas A.N., Root R.A., Lantz R.C., Sáez A.E., Chorover J. (2018). Oxidative weathering decreases bioaccessibility of toxic metal(loid)s in PM emissions from sulfide mine tailings. GeoHealth.

[66] Li Q., Wu Z., Chu B., Zhang N., Cai S., Fang J. (2007). Heavy metals in coastal wetland sediments of the Pearl River Estuary, China. Environ. Pollut..

[67] Bai Z., Li T., Zhang S., Wang G., Xu X., Zhou W., Pan X., Pu Y., Jia Y., Yang Z. (2024). Effects of climate and geochemical properties on the chemical forms of soil Cd, Pb and Cr along a more than 4000 km transect. J. Hazard. Mater..

[68] Peters R.W. (1999). Chelant extraction of heavy metals from contaminated soils. J. Hazard. Mater..

[69] Pan Y., Ye H., Yang Y., Yang C., Li X., Ma T., Dang Z., Lu G. (2023). Transport and fate of Cu and Cd in contaminated paddy soil under acid mine drainage. J. Environ. Manag..

[70] Zhao J., Deng F., Zhang H., Gu W., Chen H., Huang S. (2023). Experimental study on the redistribution law of heavy metals in sludge disintegration. J. Environ. Manag..

[71] Junusbekov M.M., Akbasova A.D., Seidakbarova A.D., Koishiyeva G.Z., Sainova G.A. (2023). Ecological assessment of soil contamination by heavy metals affected in the past by the lead-zinc mining and processing complex in Kentau, Kazakhstan. Environ. Monit. Assess..

[72] Ke W., Zeng J., Zhu F., Luo X., Feng J., He J., Xue S. (2022). Geochemical partitioning and spatial distribution of heavy metals in soils contaminated by lead smelting. Envron. Pollut..

[73] He J., Li C., Tan X., Peng Z., Li H., Luo X., Tang L., Wei J., Tang C., Yang W. (2024). Driving factors for distribution and transformation of heavy metals speciation in a zinc smelting site. J. Hazard. Mater..

[74] Du Laing G., Rinklebe J., Vandecasteele B., Meers E., Tack F.M. (2009). Trace metal behaviour in estuarine and riverine floodplain soils and sediments: A review. Sci. Total Environ..

[75] Zeng S., Li J., Wei D., Ma Y. (2017). A new model integrating short- and long-term aging of copper added to soils. PLoS ONE.

[76] Huang B., Li Z., Huang J., Chen G., Nie X., Ma W., Yao H., Zhen J., Zeng G. (2015). Aging effect on the leaching behavior of heavy metals (Cu, Zn, and Cd) in red paddy soil. Environ. Sci. Pollut. Res. Int..

[77] Zhao W., Gu C., Ying H., Feng X., Zhu M., Wang M., Tan W., Wang X. (2021). Fraction distribution of heavy metals and its relationship with iron in polluted farmland soils around distinct mining areas. Appl. Geochem..

[78] Wei H.B., Luo M., Xiang L., Zha L.S., Yang H.L. (2023). Analysis on the Distribution Characteristics and Influence Mechanism of Migration and Transformation of Heavy Metals in Mining Wasteland. Huan Jing Ke Xue.

[79] Zheng S., Zhang M. (2011). Effect of moisture regime on the redistribution of heavy metals in paddy soil. J. Environ. Sci..

[80] Yang Z., Chang J., Li X., Zhang K., Wang Y. (2022). The Effects of the Long-Term Freeze–Thaw Cycles on the Forms of Heavy Metals in Solidified/Stabilized Lead–Zinc–Cadmium Composite Heavy Metals Contaminated Soil. Appl. Sci..

[81] Wang Y., Zhang Z., Li Y., Liang C., Huang H., Wang S. (2024). Available heavy metals concentrations in agricultural soils: Relationship with soil properties and total heavy metals concentrations in different industries. J. Hazard. Mater..

[82] Zheng K., Li H., Xu L., Li S., Wang L., Wen X., Liu Q. (2019). The influence of humic acids on the weathering of pyrite: Electrochemical mechanism and environmental implications. Envron. Pollut..

[83] Liu Q., Huang Y., Zhou Y., Chen Z., Luo J., Yan X. (2023). Impacts of wet-dry alternations on cadmium and zinc immobilisation in soil remediated with iron oxides. J. Environ. Manag..

[84] Shi M., Min X., Ke Y., Lin Z., Yang Z., Wang S., Peng N., Yan X., Luo S., Wu J. (2021). Recent progress in understanding the mechanism of heavy metals retention by iron (oxyhydr)oxides. Sci. Total Environ..

[85] Zheng Y., Pan Y., Wang Z., Jiang F., Wang Y., Yi X., Dang Z. (2024). Temporal and spatial evolution of different heavy metal fractions and correlation with environmental factors after prolonged acid mine drainage irrigation: A column experiment. Sci. Total Environ..

[86] Sebola Tshisikhawe C., Ngole-Jeme V.M. (2024). Temperature induced changes on heavy metal geochemical partitioning and mobility in contaminated and uncontaminated soils. Environ. Pollut. Bioavailab..

[87] Shang G., Wang X., Zhu L., Liu S., Li H., Wang Z., Wang B., Zhang Z. (2022). Heavy Metal Pollution in Xinfengjiang River Sediment and the Response of Fish Species Abundance to Heavy Metal Concentrations. Int. J. Environ. Res. Public Health.

[88] Hou R., Zhu B., Wang L., Gao S., Wang R., Hou D. (2024). Mechanism of clay mineral modified biochar simultaneously immobilizes heavy metals and reduces soil carbon emissions. J. Environ. Manag..

[89] Shen Z., Feng Y., Yang L., Xu J., Sun Z., Luo X., Shi H. (2026). Assessing and projecting the potential cumulative risks of heavy metal dispersion (PCR-HMD) from lead-zinc mines in China: Impacts of soil water erosion and climate change. J. Hazard. Mater..

[90] Wu J., Li J., Teng Y., Chen H., Wang Y. (2020). A partition computing-based positive matrix factorization (PC-PMF) approach for the source apportionment of agricultural soil heavy metal contents and associated health risks. J. Hazard. Mater..

[91] Wang C., Chen Y., Xie Y., Feng X., Wu K., Li X., Wu P. (2025). Multi-source apportionment of soil heavy metals and spatial heterogeneity of associated risks in overlapping zones with high geological background and mining-smelting activities. Environ. Pollut..

[92] Brown S.G., Eberly S., Paatero P., Norris G.A. (2015). Methods for estimating uncertainty in PMF solutions: Examples with ambient air and water quality data and guidance on reporting PMF results. Sci. Total Environ..

[93] Liu J., Kang H., Tao W., Li H., He D., Ma L., Tang H., Wu S., Yang K., Li X. (2023). A spatial distribution-Principal component analysis (SD-PCA) model to assess pollution of heavy metals in soil. Sci. Total Environ..

[94] Li X., Di Y., Wu H., Wu H., Liu W., Luo J., Li L.J., Fan B., Zhang L.L., Li Y. (2026). Pollution characteristics and source apportionment of heavy metals in farmland soils affected by historical and ongoing mining activities through an integrated approach. J. Soils Sediments.

[95] Chen Z., Ding Y., Jiang X., Duan H., Ruan X., Li Z., Li Y. (2022). Combination of UNMIX, PMF model and Pb-Zn-Cu isotopic compositions for quantitative source apportionment of heavy metals in suburban agricultural soils. Ecotoxicol. Environ. Saf..

[96] Xiang Q., Yu H., Chu H., Hu M., Xu T., Xu X., He Z. (2022). The potential ecological risk assessment of soil heavy metals using self-organizing map. Sci. Total Envron..

[97] Zou H., Ren B. (2023). Analyzing topsoil heavy metal pollution sources and ecological risks around antimony mine waste sites by a joint methodology. Ecol. Indic..

[98] Qin Z., Peng Q., Jin C., Xu J., Xing S., Zhu P., Yang G. (2025). Geographically weighted random forest fusing multi-source environmental covariates for spatial prediction of soil heavy metals. Environ. Pollut..

[99] Kim D.M., Kwon H.L., Im D.G. (2023). Determination of contamination sources and geochemical behaviors of metals in soil of a mine area using Cu, Pb, Zn, and S isotopes and positive matrix factorization. J. Hazard. Mater..

[100] Yang S., Sun L., Sun Y., Song K., Qin Q., Zhu Z., Xue Y. (2023). Towards an integrated health risk assessment framework of soil heavy metals pollution: Theoretical basis, conceptual model, and perspectives. Environ. Pollut..

[101] Maleknia P., Mazhari S.A., Ugwonoh N., Czigány S., Kasina M., Myovela J.L. (2025). Assessment of the concentration, mobility, and bioavailability of Co, Cr, and Ni in soils from west Sabzevar ophiolitic complex, Iran. J. Asian Earth Sci. X.

[102] Wang C.C., Zhang Q.C., Kang S.G., Li M.Y., Zhang M.Y., Xu W.M., Xiang P., Ma L.Q. (2023). Heavy metal(loid)s in agricultural soil from main grain production regions of China: Bioaccessibility and health risks to humans. Sci. Total Environ..

[103] Liu X., He Y., Guo G., Liu X., Cao Y. (2026). Spatiotemporal distribution of soil heavy metals and source-specific probabilistic risk assessment in arid gold mining cluster areas of northwest China. J. Environ. Sci..

[104] Wang J., Wang B., Zhao Q., Cao J., Xiao X., Zhao D., Chen Z., Wu D. (2025). Sources analysis and risk assessment of heavy metals in soil in a polymetallic mining area in southeastern Hubei based on Monte Carlo simulation. Ecotoxicol. Environ. Saf..

[105] Yang T., Lu L., Zhou Y., Du H. (2025). Revisiting soil screening levels for heavy metal(loid)s (Cd, As, Pb) in agricultural areas: Integrating bioaccessibility and dual exposure pathways via crop consumption and soil ingestion. J. Hazard. Mater..

[106] Xiao M., Qian L., Yang B., Zeng G., Ren S. (2024). Risk assessment of heavy metals in agricultural soil based on the coupling model of Monte Carlo simulation-triangular fuzzy number. Environ. Geochem. Health.

[107] Xu L., Zhao F., Xing X., Peng J., Wang J., Ji M., Li B.L. (2024). A Review on Remediation Technology and the Remediation Evaluation of Heavy Metal-Contaminated Soils. Toxics.

[108] Song P., Xu D., Yue J., Ma Y., Dong S., Feng J. (2022). Recent advances in soil remediation technology for heavy metal contaminated sites: A critical review. Sci. Total Environ..

[109] Deo L., Osborne J.W., Benjamin L.K. (2024). Harnessing microbes for heavy metal remediation: Mechanisms and prospects. Environ. Monit. Assess..

[110] Najeeb S., Deng X., Khan R.A.A., Wu C., Wu Y. (2026). Humus-reducing bacteria in heavy metal bioremediation: Mechanisms, challenges, and prospects. Ecotoxicol. Environ. Saf..

[111] Mao J., Li J., Shuai H., Zhang Y., Wang Y., Yu X. (2026). Multidimensional interaction mechanisms and synergistic remediation strategies for heavy metal with co-occurring pollutants: A review. Ecotoxicol. Environ. Saf..

[112] Karimpour M., Ashrafi S.D., Taghavi K., Mojtahedi A., Roohbakhsh E., Naghipour D. (2018). Adsorption of cadmium and lead onto live and dead cell mass of *Pseudomonas aeruginosa*: A dataset. Data Brief..

[113] Gorny J., Billon G., Noiriel C., Dumoulin D., Lesven L., Madé B. (2016). Chromium behavior in aquatic environments: A review. Environ. Rev..

[114] Peng L., Liu Y., Gao S.H., Dai X., Ni B.J. (2015). Assessing chromate reduction by dissimilatory iron reducing bacteria using mathematical modeling. Chemosphere.

[115] Ouyang X., Guo Z., Bian L., Dang Z., Xu M., Yin H. (2026). Simultaneous stabilization of As/Pb and enhancement of nitrogen retention in farmland soil by the novel Fe-Mn oxidizing bacteria co-inoculation. J. Hazard. Mater..

[116] Elhaj Baddar Z., Xu X., Spencer B. (2023). Spatiotemporal Changes in Trace Metal Bioavailability in the Sediment Pore water of a Constructed Wetland Using Passive Pore water Samplers. Environ. Toxicol. Chem..

[117] Shaji P.S., Vincent S.G.T., Subburamu K. (2025). Sulfate-reducing bacteria in removal of pollutants: A promising candidate for bioremediation. World J. Microbiol. Biotechnol..

[118] Azab E., Hegazy A.K. (2020). Monitoring the Efficiency of *Rhazya stricta* L. Plants in Phytoremediation of Heavy Metal-Contaminated Soil. Pharmaceuticals.

[119] Song W., Wang J., Zhai L., Ge L., Hao S., Shi L., Lian C., Chen C., Shen Z., Chen Y. (2022). A meta-analysis about the accumulation of heavy metals uptake by *Sedum alfredii* and *Sedum plumbizincicola* in contaminated soil. Int. J. Phytoremediation.

[120] Liu H., Zhao H., Wu L., Xu W. (2017). A Genetic Transformation Method for Cadmium Hyperaccumulator *Sedum plumbizincicola* and Non-hyperaccumulating Ecotype of *Sedum alfredii*. Front. Plant Sci..

[121] Li J.T., Gurajala H.K., Wu L.H., van der Ent A., Qiu R.L., Baker A.J.M., Tang Y.T., Yang X.E., Shu W.S. (2018). Hyperaccumulator Plants from China: A Synthesis of the Current State of Knowledge. Environ. Sci. Technol..

[122] Zhu X., Tu C., Zhou J., Yang S., Li Y., Wu L., Newman L.A., Luo Y. (2025). Cadmium phytoextraction by *Sedum alfredii* and *Sedum plumbizincicola*: Mechanisms, challenges and prospects. Int. J. Phytorem..

[123] Zhao F., Han Y., Shi H., Wang G., Zhou M., Chen Y. (2023). Arsenic in the hyperaccumulator Pteris vittata: A review of benefits, toxicity, and metabolism. Sci. Total Environ..

[124] Cui S., Xiao H., Miao D., Yang W. (2023). Metal uptake and translocation by Chinese brake fern (Pteris vittata) and diversity of rhizosphere microbial communities under single and combined arsenic and cadmium stress. Environ. Sci. Pollut. Res. Int..

[125] Acharya A., Bellaloui N., Pilipovic A., Perez E., Maddox-Mandolini M., Fuente H. (2025). Current Assessment and Future Perspectives on Phytoremediation of Heavy Metals. Pharmaceuticals.

[126] Li X., Zhang X., Yang Y., Li B., Wu Y., Sun H., Yang Y. (2016). Cadmium Accumulation Characteristics in Turnip Landraces from China and Assessment of Their Phytoremediation Potential for Contaminated Soils. Front. Plant Sci..

[127] Teng D., Mao K., Ali W., Xu G., Huang G., Niazi N.K., Feng X., Zhang H. (2020). Describing the toxicity and sources and the remediation technologies for mercury-contaminated soil. RSC Adv..

[128] Durante-Yánez E.V., Martínez-Macea M.A., Enamorado-Montes G., Combatt Caballero E., Marrugo-Negrete J. (2022). Phytoremediation of Soils Contaminated with Heavy Metals from Gold Mining Activities Using *Clidemia sericea* D. Don. Pharmaceuticals.

[129] Balint R., Boajă I.P. (2024). Assisted phytoextraction as a nature-based solution for the sustainable remediation of metal(loid)-contaminated soils. Integr. Environ. Assess. Manag..

[130] Santa-Cruz J., Robinson B., Krutyakov Y.A., Shapoval O.A., Peñaloza P., Yáñez C., Neaman A. (2023). An Assessment of the Feasibility of Phytoextraction for the Stripping of Bioavailable Metals from Contaminated Soils. Environ. Toxicol. Chem..

[131] Mesa-Marín J., Pérez-Romero J.A., Redondo-Gómez S., Pajuelo E., Rodríguez-Llorente I.D., Mateos-Naranjo E. (2020). Impact of Plant Growth Promoting Bacteria on *Salicornia ramosissima* Ecophysiology and Heavy Metal Phytoremediation Capacity in Estuarine Soils. Front. Microbiol..

[132] Yu X., Zhang T., Guo J., Ma T., Shang J., Huang Y., Liu Y. (2025). Plants colonization accelerates galena oxidation, mineralogical transformation, and microbial community reshaping under the soil phytoremediation processes. Environ. Res..

[133] Dovletyarova E.A., Slukovskaya M.V., Ivanova T.K., Mosendz I.A., Novikov A.I., Chaporgina A.A., Soshina A.S., Myazin V.A., Korneykova M.V., Ettler V. (2024). Sensitivity of microbial bioindicators in assessing metal immobilization success in smelter-impacted soils. Chemosphere.

[134] Zhang S., Li T., Zhang X., Yu H., Zheng Z., Wang Y., Hao X., Pu Y. (2014). Changes in pH, dissolved organic matter and Cd species in the rhizosphere soils of Cd phytostabilizer Athyrium wardii (Hook.) Makino involved in Cd tolerance and accumulation. Environ. Sci. Pollut. Res. Int..

[135] Radziemska M., Klik B. (2025). Biochar-Assisted Phytostabilization of Heavy Metal-Polluted Land. Plantation-Based Land Restoration.

[136] Pinna M.V., Diquattro S., Garau M., Grottola C.M., Giudicianni P., Roggero P.P., Castaldi P., Garau G. (2024). Combining biochar and grass-legume mixture to improve the phytoremediation of soils contaminated with potentially toxic elements (PTEs). Heliyon.

[137] Cheng S., Chen T., Xu W., Huang J., Jiang S., Yan B. (2020). Application Research of Biochar for the Remediation of Soil Heavy Metals Contamination: A Review. Molecules.

[138] Liu J., Ji Y., Xie W., Yang J., Wang X., Feng Y., Yu X., Yao R., Yang J.R., Zhu H. (2026). Synergistic suppression of ammonia volatilization by biochar-compost through soil physicochemical and microbial regulation in saline-alkali soil. J. Environ. Manag..

[139] Kumar S., Kumar R., Sindhu S.S. (2026). Biochar and biofertilizers combined fertilization technology for modulating soil health, plant growth and environmental sustainability. Total Environ. Microbiol..

[140] Ge H., Sui F., Pu Y., Quan G., Cui L., Yan J. (2025). Effects of different biochars on Cd immobilization in rice paddy soil: A two-year field study. BioResources.

[141] Cui L., Li L., Zhang A., Pan G., Bao D., Chang A. (2011). Biochar amendment greatly reduces rice Cd uptake in a contaminated paddy soil: A two-year field experiment. Bioresources.

[142] Huang W., Jia Y., Niu C., Zhang H., Wang Y., Feng C. (2024). Effects of carbon-based modified materials on soil water and fertilizer retention and pollution control in rice root zone. Sustainability.

[143] Li X., Huang D., Huang H., Wang G., Xu W., Lei Y., Zhou W. (2026). Mechanistic insights into antibiotic resistance control by nano zero-valent iron (nZVI) and modified nZVI: Interfacial reaction and the role of in-situ generated iron oxides. J. Hazard. Mater..

[144] Cui X., Hou D., Tang Y., Liu M., Qie H., Qian T., Xu R., Lin A., Xu X. (2023). Effects of the application of nanoscale zero-valent iron on plants: Meta analysis, mechanism, and prospects. Sci. Total Environ..

[145] Zhang Y., Zhang Y., Wu A. (2024). Design and construction of magnetic nanomaterials and their remediation mechanisms for heavy metal contaminated soil. Sci. Total Environ..

[146] Umair M., Huma Zafar S., Cheema M., Usman M. (2024). New insights into the environmental application of hybrid nanoparticles in metal contaminated agroecosystem: A review. J. Environ. Manag..

[147] Wu L., Yang X., Zhang F., Zhang Z. (2024). Effects of biochar-supported nano-hydroxyapatite on cadmium availability and pepper growth in contaminated soils. Sci. Total Environ..

[148] Wu W., Ye W., He L., Wu M., Li J., Yue Z., Deng R. (2025). The restoration of zinc pollution in smelting site soil using nanohydroxyapatite-modified cyanobacterial biochar and its mechanism. Environ. Res..

[149] Yuanyuan L., Zhuo Z. (2026). Global Systematic Review of Sustainability Assessment for Contaminated Site Remediation: A Bibliometric Visualization Approach. J. Chem. Chem. Eng..

[150] Azuazu I.N., Sam K., Campo P., Coulon F. (2023). Challenges and opportunities for low-carbon remediation in the Niger Delta: Towards sustainable environmental management. Sci. Total Environ..

[151] Masud M.A.A., Shin W.S., Sarker A., Septian A., Das K., Deepo D.M., Iqbal M.A., Islam A.R.M.T., Malafaia G. (2023). A critical review of sustainable application of biochar for green remediation: Research uncertainty and future directions. Sci. Total Environ..

[152] Alshehri K., Gao Z., Harbottle M., Sapsford D., Cleall P. (2023). Life cycle assessment and cost-benefit analysis of nature-based solutions for contaminated land remediation: A mini-review. Heliyon.

