# Supplementary material for: Integrated Assessment of Heavy Metals in Mining-Affected Soils in China: Source Identification, Ecological Risks, and Sustainable Remediation
Source: Toxics. 2026 Aug 4;14(8):689. doi: 10.3390/toxics14080689 (PMC13517496; doi:10.3390/toxics14080689)
Supplement: Supplementary file 1 [file toxics-14-00689-s001.zip › toxics-4408019-supplementary.pdf]

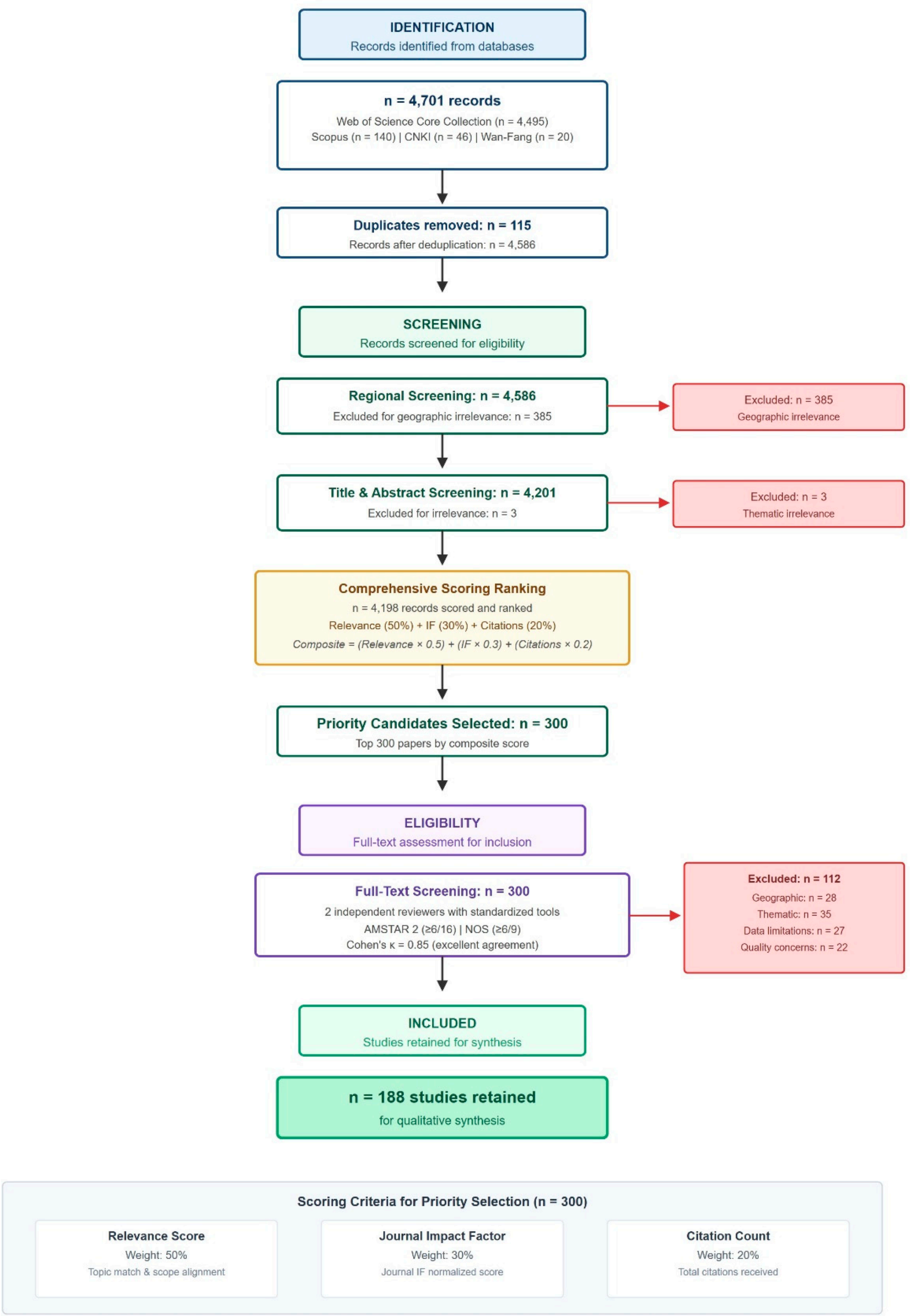

Figure S1. PRISMA 2020 Flowchart of Literature Search and Screening Process

Table S1. Summary of All 152 Included Studies

Reference	Authors	Year	Study Area	Mining Type	Main Metals	Methodology	Principal Findings
[1]	Xie, S. et al.	2025	China	Non-ferrous metallurgical	Multiple	Review	Overview and prospects of modeling and optimal control for non-ferrous metallurgical processes and mineral processing
[2]	Zhou, Y. et al.	2024	Gold smelting area, China	Gold mining	As, Cd, Pb, Hg	Source-oriented risk assessment, PMF	Tailings contributed 60% of ecological risk; traffic sources dominated human health risk
[3]	Ha, N. T. H. et al.	2026	Global	Metal mining	Multiple	Review, meta-analysis	Comprehensive review of heavy metal accumulation, sources, food safety, and health risks at metal mines
[4]	Shi, J. et al.	2025	Ultramafic rock area, China	High geological background	High-backgroun d elements	Geochemical characterization	Distribution characteristics and activity assessment of high-background elements
[5]	Zhao, X. et al.	2023	China	Metal mining	Multiple	Review	Comprehensive review on heavy metal pollution and remediation technology in mining areas
[6]	Yang, W. et al.	2024	Kangdian metallogenic province, Yunnan	Pb-Zn mining	Cd, Pb, Zn, Cu	Machine learning	Widespread contamination with strong spatial heterogeneity
[7]	Xu, D. M. et al.	2021	China	Smelter contaminated	Multiple	Review	Critical review of smelter-contaminated soils, health risks, and remediation progress
[8]	Wei, N. et al.	2025	Karst region, southwestern China	High geological background	Cd, Ni, Zn	Geochemical speciation	Natural high background combined with mining creates complex pollution

[9]	Xu, S. et al.	2025	Northwestern China (Shaanxi, Gansu)	Cu/Mo mining	Cu, Cd, Pb	Ecological risk assessment	AMD-induced acidification accelerates heavy metal mobilization
[10]	Yan, Y. et al.	2025	Henan Province	Agricultural soil near mines	Cd, Pb, As, Hg	Health risk assessment	32% rice samples exceeded Cd safety standard; HI > 1 for children and adults
[11]	Deng, M. et al.	2020	Farmland soil, China	Agricultural soil near mines	Multiple	Source apportionment, transfer prediction	Metals source apportionment and prediction of soil-rice-human chain transfer
[12]	Landrigan, P. J. et al.	1981	Global	Smelter exposure	Multiple	Epidemiology	Epidemiology and toxic consequences of heavy metal exposure in children
[13]	Sun, Y. et al.	2025	Wuzhou, Guangxi	Rare/precious metal mining	As, Cd, Pb	Characterization + microbiological analysis	Combined pollution risk with significant microbial community impacts
[14]	Song, L. et al.	2026	Central Yunnan Dry-Hot Valley	Agricultural soil near mines	Cd, Pb, As	Risk assessment + source apportionment	Heavy metal accumulation with significant ecological and health risks
[15]	Xu, Y. et al.	2022	Hulan River Watershed, Northeastern China	Agricultural soil near mines	Cd, Pb, Cu, Zn	PMF, SOM, GIS	Multi-model integration enables comprehensive source apportionment and risk mapping
[16]	Lv, X. et al.	2025	Mining area, China	Metal mining	Cd, Pb, Cu, Zn	SOM-PMF coupled model	SOM-PMF improves source apportionment accuracy in high heterogeneity areas
[17]	Lin, Y. et al.	2026	Chinese industrialized bay	Multi-media pollution	Trace metals	SOM, PMF, GIS	Source apportionment and ecological risks in multiple media

[18]	Wu, Y. et al.	2024	Agricultural soils near mining areas	Multi-source pollution	Cd, Pb, As, Cu	Multi-receptor modeling + Monte Carlo	Probabilistic approach reveals 23.5% probability of HI > 1 for children
[19]	Ma, J. et al.	2023	Farmland soil, China	Agricultural soil near mines	Multiple	Three receptor models	Source apportionment and model applicability comparison
[20]	Yuan, B. et al.	2023	Typical mining city, China	Metal mining	Cd, Pb, As, Hg	Source-oriented probabilistic risk assessment	Mining sources contribute most to total health risk
[21]	Xie, G. et al.	2025	Eastern China	Selenium-enriched soils	Multiple	Enrichment and source apportionment	Heavy metal enrichment in selenium-enriched soils
[22]	Huang, W. et al.	2025	Arsenic waste mine, Yunnan	As mining	As, Cd, Pb	Source-specific risk assessment	Integrated environmental and biological factors improve risk characterization
[23]	Ni, M. et al.	2025	Chengdu Plain	Urban agricultural soils	Multiple	Integrated multi-model approach	Identifying priority control factors for heavy metal pollution
[24]	Sen, T. K.	2023	Global	Water/wastewater	Multiple	Review	Review of agricultural solid waste-based adsorbents for heavy metal remediation
[25]	Wang, L. et al.	2021	Global	Heavy metal contaminated soil	Multiple	Review	Review of green remediation strategies for heavy metal contaminated soil
[26]	Yang, S. et al.	2024	Cd-contaminated farmland	Agricultural soil	Cd	Plant-microbe technology	Effects of Priestia aryabhattai on phosphorus fraction and Cd remediation
[27]	Raklami, A. et al.	2022	Global	Heavy metal contaminated soil	Multiple	Review	Plants-microorganisms-based bioremediation for heavy metal cleanup
[28]	Saxena, G. et al.	2020	Global	Heavy metal contaminated sites	Multiple	Review	Phytoremediation of heavy metal-contaminated sites: eco-environmental concerns

[29]	Zheng, K. et al.	2023	Heavy metal contaminated soils	Agricultural soil	Multiple	Microbial inoculants + legumes	Enhancing remediation potential through synergistic application of microbial inoculants and legumes
[30]	Krishna Raj, C. et al.	2025	Global	Environmental restoration	Multiple	Review	Bioremediation: recent progress, challenges, and future directions
[31]	Firincă, C. et al.	2025	Global	Heavy metal contaminated soil	Multiple	Review	Innovative approaches and evolving strategies in heavy metal bioremediation
[32]	Jha, A. et al.	2024	Agricultural soil	Agricultural soil	Multiple	Nano-biogenic remediation	Nano-biogenic heavy metal adsorptive remediation for enhanced soil health
[33]	Wang, X. et al.	2025	Zijiang River, Hunan	Antimony ore	Sb, multiple	Pollution characteristics, risk assessment	Pollution characteristics and source comparative analysis of antimony ore areas
[34]	García, G. et al.	2025	Abandoned metal mining district	Abandoned mine	Multiple	Environmental pollution assessment	Underlying causes of long-term environmental pollution by mine waste
[35]	Xu, M. et al.	2026	Multi-source composite pollution area	Multi-source pollution	Multiple	PMF + health risk + spatialized emission inventories	Causal inference-driven framework for source verification
[36]	Li, P. et al.	2015	Lead/zinc smelter, southwestern China	Pb-Zn smelting	Cd, Pb, Zn, Cu	Contamination and health risk assessment	Contamination and health risks of soil heavy metals around lead/zinc smelter
[37]	Yang, L. et al.	2024	China	Agricultural soil	Multiple	Meta-analysis	Analysis and evaluation of heavy metal pollution in farmland soil

[38]	Meng, X. F. et al.	2021	Henan Province	Industrial area	Multiple	Spatial distribution and risk assessment	Spatial distribution and risk assessment of heavy metal pollution
[39]	Xia, B. et al.	2025	Mining cities	Mining pollution	Cd, multiple	Cd isotopes + multivariate statistical method	Identifying hidden heavy metal sources in atmospheric dust
[40]	Zhang, J. L. et al.	2021	Southwest China	Metal mining	Multiple	Meta-analysis	Effects of metal mining on soil heavy metal concentrations in Southwest China
[41]	Zhu, D. et al.	2025	Sichuan Province	Mining area	Multiple	Contamination assessment and health risks	Assessment of heavy metal contamination levels and health risks
[42]	Hui, J. et al.	2026	China	Mining area	Multiple	Pollution-enhanced remote sensing ecological index	Integrating soil heavy metal risk into ecological quality assessment
[43]	Jin, H. et al.	2023	Abandoned zinc smelting site	Zn smelting	Multiple	GIS and PMF models	Source apportionment and quantitative risk assessment at zinc smelting site
[44]	Chen, R. et al.	2022	Southern Shaanxi	Mining area	Multiple	Pollution assessment and health risks	Assessment of soil-heavy metal pollution and health risks
[45]	Jiang, Y. et al.	2025	Huang-Huai-Hai Plain, Henan	Agricultural soil	Multiple	Background values assessment	Background values of soil heavy metals in Henan Province
[46]	Li, Q. et al.	2023	Niujiaotang, China	Pb-Zn mine tailing	Pb, Zn	Risk assessment	Risk assessment of heavy metals in farmlands near Pb-Zn mine tailing ponds
[47]	Hu, F. et al.	2026	Pb-Zn mine area	Pb-Zn mining	Multiple	Ecotoxicological effects and health risk assessment	Ecotoxicological effects of heavy metals on rice and health risk assessment

[48]	Zang, Z. et al.	2020	China	Pb/Zn smelting	Pb, Zn	Spatiotemporal variation and pollution assessment	Spatiotemporal variation and pollution assessment of Pb/Zn from smelting activities
[49]	Hua, L. et al.	2018	Typical mineral mining city, China	Mineral mining	Multiple	Spatial distributions, pollution assessment, and source apportionment	Spatial distributions and pollution assessment in mineral mining city
[50]	Jin, L. et al.	2026	Smelting and mining area	Smelting/mining	Multiple	Sources, flux, and potential risks	Sources, flux, and potential risks of heavy metals around smelting and mining area
[51]	Van Alphen, M.	1999	Lead-zinc smelter area	Pb-Zn smelting	Pb, Zn, Cd	Atmospheric deposition plumes	Atmospheric heavy metal deposition plumes adjacent to primary lead-zinc smelter
[52]	Guo, G. et al.	2025	Soil pollution area	Multiple sources	Multiple	Probabilistic source-specific eco-health risk assessment	Prioritizing pollution sources and targeted potentially toxic elements
[53]	Hou, Y. et al.	2023	Global	Gold mining	Multiple	Global meta-analysis	Environmental contamination and health risk assessment of potentially toxic elements near gold mines
[54]	González-Valoys, A. C. et al.	2022	Panama	Gold mining	Multiple	Environmental challenges	Environmental challenges related to cyanidation in Central American gold mining
[55]	Chen, M. H. et al.	2022	Hainan Province	Gold mining	Multiple	Pollution tracing and human risks	Tracing pollution and human risks of potentially toxic elements near gold mine

[56]	Bai, J. et al.	2022	Rare earth mining areas, China	Rare earth mining	Multiple	Resource and environmental carrying capacity evaluation	Evaluation of resource and environmental carrying capacity in rare earth mining areas
[57]	Wang, L. et al.	2016	Inner Mongolia	Rare earth mining	Rare earth elements	Anomalous abundance and redistribution patterns	Anomalous abundance and redistribution patterns of rare earth elements
[58]	Guo, Z. et al.	2025	Ionic rare earth mining areas	Rare earth mining	Zn	Release pattern study	Study on the release pattern of Zn in ionic rare earth mining area soils
[59]	Götzke, L. et al.	2019	Global	F-block metal chemistry	F-block metals	Coordination chemistry review	Coordination chemistry of f-block metal ions with bio-relevant functional groups
[60]	Adnan, M. et al.	2025	Abandoned Pb/Zn smelting sites	Pb/Zn smelting	Multiple	Pollution and source analysis	Heavy metal pollution and source analysis around abandoned Pb/Zn smelting sites
[61]	Chrastný, V. et al.	2012	Olkusz mine/smelter, Poland	Pb/Zn mining/smelting	Pb, Zn, Cd	Geochemical position analysis	Geochemical position of Pb, Zn and Cd in soils near mine/smelter
[62]	Zhu, Y. et al.	2025	Chinese megacity	Non-ferrous metal mining	Multiple	PM2.5-bound heavy metals	Source-specific health risks of PM2.5-bound heavy metals
[63]	Luo, X. et al.	2023	Pb/Zn smelting regions, China	Pb/Zn smelting	Multiple	Pollution assessment and remediation potential	Soil heavy metal pollution and remediation potential of biomineralization
[64]	Zhou, L. et al.	2023	Watershed scale	Heavy metal transport	Multiple	Transport and fate modeling	Modeling transport and fate of heavy metals at watershed scale

[65]	Thomas, A. N. et al.	2018	Sulfide mine tailings	Mining waste	Multiple	Oxidative weathering	Oxidative weathering decreases bioaccessibility of toxic metal(loid)s
[66]	Li, Q. et al.	2007	Pearl River Estuary	Coastal wetland	Multiple	Heavy metal analysis	Heavy metals in coastal wetland sediments
[67]	Bai, Z. et al.	2024	China (4000 km transect)	Geochemical survey	Cd, Pb, Cr	Climate and geochemical properties effects	Effects of climate and geochemical properties on chemical forms
[68]	Peters, R. W.	1999	Contaminated soil	Chemical remediation	Multiple	Chelant extraction	Chelant extraction of heavy metals from contaminated soils
[69]	Pan, Y. et al.	2023	Contaminated paddy soil	Acid mine drainage	Cu, Cd	Transport and fate study	Transport and fate of Cu and Cd under acid mine drainage
[70]	Zhao, J. et al.	2023	Sludge	Heavy metal redistribution	Multiple	Sludge disintegration study	Experimental study on heavy metal redistribution in sludge disintegration
[71]	Junusbekov, M. M. et al.	2023	Kentau, Kazakhstan	Lead-zinc mining	Multiple	Ecological assessment	Ecological assessment of soil contamination by heavy metals
[72]	Ke, W. et al.	2022	Lead smelting area	Pb smelting	Multiple	Geochemical partitioning	Geochemical partitioning and spatial distribution of heavy metals
[73]	He, J. et al.	2024	Zinc smelting site	Zn smelting	Multiple	Driving factors for speciation distribution	Driving factors for distribution and transformation of heavy metal speciation
[74]	Du Laing, G. et al.	2009	Estuarine and riverine floodplain	Natural/sediment pollution	Multiple	Trace metal behavior review	Trace metal behaviour in estuarine and riverine floodplain soils
[75]	Zeng, S. et al.	2017	Copper-contaminated soil	Cu pollution	Cu	Aging effect study	New model integrating short- and long-term aging of copper

[76]	Huang, B. et al.	2015	Red paddy soil	Cu, Zn, Cd pollution	Cu, Zn, Cd	Aging effect on leaching behavior	Aging effect on leaching behavior of heavy metals
[77]	Zhao, W. et al.	2021	Polluted farmland around mining areas	Mining pollution	Multiple	Fraction distribution	Fraction distribution of heavy metals and relationship with iron
[78]	Wei, H. B. et al.	2023	Mining wasteland	Mining pollution	Multiple	Migration and transformation analysis	Analysis on distribution characteristics and migration mechanism
[79]	Zheng, S. et al.	2011	Paddy soil	Heavy metal pollution	Multiple	Moisture regime effect	Effect of moisture regime on heavy metal redistribution
[80]	Yang, Z. et al.	2022	Pb-Zn-Cd composite contaminated soil	Solidified/stabilized soil	Pb, Zn, Cd	Freeze-thaw cycles effect	Effects of freeze-thaw cycles on heavy metal forms
[81]	Wang, Y. et al.	2024	Agricultural soils	Multiple industries	Multiple	Available heavy metals analysis	Available heavy metal concentrations and relationship with soil properties
[82]	Zheng, K. et al.	2019	Pyrite weathering	Mining waste	Multiple	Humic acids influence	Influence of humic acids on pyrite weathering
[83]	Liu, Q. et al.	2023	Remediated soil	Iron oxides remediation	Cd, Zn	Wet-dry alternations effect	Impacts of wet-dry alternations on cadmium and zinc immobilisation
[84]	Shi, M. et al.	2021	Iron (oxyhydr)oxides	Natural/synthetic materials	Multiple	Heavy metal retention mechanism	Recent progress in understanding heavy metal retention by iron oxides
[85]	Zheng, Y. et al.	2024	Acid mine drainage irrigation	AMD pollution	Multiple	Temporal and spatial evolution	Temporal and spatial evolution of heavy metal fractions under AMD irrigation

[86]	Sebola Tshisikhawe, C. et al.	2024	Contaminated and uncontaminated soils	Various	Multiple	Temperature effects	Temperature induced changes on heavy metal geochemical partitioning
[87]	Shang, G. et al.	2022	Xinfengjiang River	River sediment	Multiple	Heavy metal pollution and fish response	Heavy metal pollution in river sediment and fish species response
[88]	Hou, R. et al.	2024	Heavy metal contaminated soil	Biochar remediation	Multiple	Clay mineral modified biochar	Mechanism of clay mineral modified biochar immobilizing heavy metals
[89]	Shen, Z. et al.	2026	Lead-zinc mines, China	Pb-Zn mining	Multiple	Cumulative risk projection	Assessing and projecting potential cumulative risks of heavy metal dispersion
[90]	Wu, J. et al.	2020	Agricultural soil	Multi-source pollution	Multiple	PC-PMF approach	Partition computing-based positive matrix factorization for source apportionment
[91]	Wang, C. et al.	2025	Overlapping zones	High geological background + mining	Multiple	Multi-source apportionment	Multi-source apportionment in overlapping zones with high geological background
[92]	Brown, S. G. et al.	2015	Ambient air/water	Environmental monitoring	Multiple	PMF uncertainty estimation	Methods for estimating uncertainty in PMF solutions
[93]	Liu, J. et al.	2023	Soil pollution	Multiple sources	Multiple	SD-PCA model	Spatial distribution-principal component analysis model
[94]	Li, X. et al.	2026	Farmland soil affected by mining	Historical/ongoing mining	Multiple	Integrated approach	Pollution characteristics and source apportionment through integrated approach
[95]	Chen, Z. et al.	2022	Suburban agricultural soil	Multi-source pollution	Multiple	UNMIX + PMF + isotopes	Combination of UNMIX, PMF model and isotopic compositions
[96]	Xiang, Q. et al.	2022	Soil pollution	Multiple sources	Multiple	Self-organizing map	Potential ecological risk assessment using self-organizing map

[97]	Zou, H. et al.	2023	Antimony mine waste sites	Sb mining	Sb, multiple	Joint methodology	Analyzing pollution sources and ecological risks using joint methodology
[98]	Qin, Z. et al.	2025	Soil pollution	Multiple sources	Multiple	Geographically weighted random forest	Geographically weighted random forest for spatial prediction
[99]	Kim, D. M. et al.	2023	Mine area, Korea	Metal mining	Cu, Pb, Zn, S	Isotopes + PMF	Determination of contamination sources using isotopes and PMF
[100]	Yang, S. et al.	2023	Global	Soil heavy metal pollution	Multiple	Review	Towards integrated health risk assessment framework
[101]	Maleknia, P. et al.	2025	Ophiolitic complex, Iran	Natural geological background	Co, Cr, Ni	Geochemical assessment	Assessment of concentration, mobility, and bioavailability
[102]	Wang, C. C. et al.	2023	Main grain production regions, China	Agricultural soil	Multiple	Bioaccessibility and health risks	Heavy metal(loid)s bioaccessibility and health risks in agricultural soil
[103]	Liu, X. et al.	2026	Arid gold mining cluster areas, northwest China	Gold mining	Multiple	Spatiotemporal distribution and source-specific risk assessment	Spatiotemporal distribution and source-specific probabilistic risk assessment
[104]	Wang, J. et al.	2025	Polymetallic mining area, southeastern Hubei	Polymetallic mining	Multiple	Monte Carlo simulation	Sources analysis and risk assessment based on Monte Carlo simulation
[105]	Yang, T. et al.	2025	Agricultural areas	Heavy metal pollution	Cd, As, Pb	Soil screening levels revision	Revisiting soil screening levels integrating bioaccessibility

[106]	Xiao, M. et al.	2024	Agricultural soil	Heavy metal pollution	Multiple	Monte Carlo + triangular fuzzy number	Risk assessment based on coupling model of Monte Carlo simulation
[107]	Xu, L. et al.	2024	Global	Heavy metal contaminated soil	Multiple	Review	Review on remediation technology and evaluation
[108]	Song, P. et al.	2022	Global	Heavy metal contaminated sites	Multiple	Review	Recent advances in soil remediation technology
[109]	Deo, L. et al.	2024	Global	Heavy metal remediation	Multiple	Review	Harnessing microbes for heavy metal remediation
[110]	Najeeb, S. et al.	2026	Heavy metal bioremediation	Bioremediation	Multiple	Review	Humus-reducing bacteria in heavy metal bioremediation
[111]	Mao, J. et al.	2026	Heavy metal co-occurring pollutants	Multiple pollution	Multiple	Review	Multidimensional interaction mechanisms and synergistic remediation strategies
[112]	Karimpour, M. et al.	2018	Laboratory study	Microbial adsorption	Cd, Pb	Adsorption dataset	Adsorption of cadmium and lead onto Pseudomonas aeruginosa
[113]	Gorny, J. et al.	2016	Aquatic environments	Chromium behavior	Cr	Review	Chromium behavior in aquatic environments
[114]	Peng, L. et al.	2015	Chromate reduction	Microbial reduction	Cr	Mathematical modeling	Assessing chromate reduction by dissimilatory iron reducing bacteria
[115]	Ouyang, X. et al.	2026	Farmland soil	As/Pb pollution	As, Pb	Fe-Mn oxidizing bacteria	Simultaneous stabilization of As/Pb by Fe-Mn oxidizing bacteria
[116]	Elhaj Baddar, Z. et al.	2023	Constructed wetland	Trace metal bioavailability	Multiple	Passive pore water samplers	Spatiotemporal changes in trace metal bioavailability

[117]	Shaji, P. S. et al.	2025	Global	Sulfate-reducing bacteria	Multiple	Review	Sulfate-reducing bacteria in removal of pollutants
[118]	Azab, E. et al.	2020	Heavy metal-contaminated soil	Phytoremediation	Multiple	Rhazya stricta phytoremediation	Monitoring efficiency of Rhazya stricta in phytoremediation
[119]	Song, W. et al.	2022	Contaminated soil	Phytoremediation	Multiple	Meta-analysis	Accumulation of heavy metals by Sedum alfredii and Sedum plumbizincicola
[120]	Liu, H. et al.	2017	Sedum plumbizincicola	Genetic transformation	Cd	Genetic transformation method	Genetic transformation method for cadmium hyperaccumulator
[121]	Li, J. T. et al.	2018	China	Hyperaccumulator plants	Multiple	Review	Hyperaccumulator plants from China
[122]	Zhu, X. et al.	2025	Contaminated soil	Phytoremediation	Cd	Sedum alfredii and Sedum plumbizincicola	Cadmium phytoextraction by Sedum species
[123]	Zhao, F. et al.	2023	Pteris vittata	Arsenic hyperaccumulation	As	Review	Arsenic in hyperaccumulator Pteris vittata
[124]	Cui, S. et al.	2023	Arsenic and cadmium stress	Phytoremediation	As, Cd	Chinese brake fern	Metal uptake and rhizosphere microbial communities under As and Cd stress
[125]	Acharya, A. et al.	2025	Global	Phytoremediation	Multiple	Review	Current assessment and future perspectives on phytoremediation
[126]	Li, X. et al.	2016	Cadmium-contaminated soil	Phytoremediation	Cd	Turnip landraces	Cadmium accumulation characteristics in turnip landraces
[127]	Teng, D. et al.	2020	Mercury-contaminated soil	Mercury remediation	Hg	Review	Describing toxicity, sources, and remediation technologies for mercury

[128]	Durante-Yánez, E. V. et al.	2022	Gold mining activities	Phytoremediation	Multiple	Clidemia sericea	Phytoremediation using Clidemia sericea for gold mining contaminated soils
[129]	Balint, R. et al.	2024	Metal(loid)-contaminated soil	Assisted phytoextraction	Multiple	Review	Assisted phytoextraction as nature-based solution
[130]	Santa-Cruz, J. et al.	2023	Contaminated soil	Phytoextraction	Multiple	Feasibility assessment	Assessment of feasibility of phytoextraction for metal stripping
[131]	Mesa-Marín, J. et al.	2020	Estuarine soil	Phytoremediation	Multiple	Plant growth promoting bacteria	Impact of plant growth promoting bacteria on phytoremediation
[132]	Yu, X. et al.	2025	Soil phytoremediation	Phytoremediation	Multiple	Plant colonization	Plants colonization accelerates mineralogical transformation
[133]	Dovletyarova, E. A. et al.	2024	Smelter-impacted soil	Metal immobilization	Multiple	Microbial bioindicators	Sensitivity of microbial bioindicators in assessing metal immobilization
[134]	Zhang, S. et al.	2014	Cd-contaminated soil	Phytostabilization	Cd	Athyrium wardii	Changes in pH, DOM, and Cd species in phytostabilizer rhizosphere
[135]	Radziemska, M. et al.	2025	Heavy metal-polluted land	Biochar-assisted phytostabilization	Multiple	Review	Biochar-assisted phytostabilization of heavy metal-polluted land
[136]	Pinna, M. V. et al.	2024	PTE-contaminated soil	Biochar + grass-legume mixture	PTEs	Phytoremediation improvement	Combining biochar and grass-legume mixture to improve phytoremediation
[137]	Cheng, S. et al.	2020	Heavy metal contaminated soil	Biochar remediation	Multiple	Review	Application research of biochar for soil heavy metal remediation
[138]	Liu, J. et al.	2026	Saline-alkali soil	Biochar-compost	Multiple	Ammonia volatilization suppression	Synergistic suppression of ammonia volatilization by biochar-compost
[139]	Kumar, S. et al.	2026	Agricultural soil	Biochar + biofertilizers	Multiple	Soil health improvement	Biochar and biofertilizers combined fertilization technology

[140]	Ge, H. et al.	2025	Cd-contaminated paddy soil	Biochar amendment	Cd	Two-year field study	Rice straw biochar reduces soil available Cd and rice grain Cd
[141]	Cui, L. et al.	2011	Cd-contaminated paddy soil	Biochar amendment	Cd	Two-year field experiment	Biochar reduces rice grain Cd by 16.8%-61.9% and soil available Cd by 32.0%-52.5%
[142]	Huang, W. et al.	2024	Cd-contaminated soil	Carbon-based modified materials	Cd	Soil water and fertilizer retention	Carbon-based materials reduce DTPA-extractable Cd by 79.69%
[143]	Li, X. et al.	2026	Antibiotic resistance + heavy metal	nZVI remediation	Multiple	Mechanistic insights	Mechanistic insights into antibiotic resistance control by nZVI
[144]	Cui, X. et al.	2023	Heavy metal contaminated soil	nZVI application	Multiple	Meta-analysis	Effects of nZVI on plants: reduced Cd accumulation by 38.2%, Pb by 42.1%
[145]	Zhang, Y. et al.	2024	Heavy metal contaminated soil	Magnetic nanomaterials	Multiple	Design and construction	Design and construction of magnetic nanomaterials for remediation
[146]	Umair, M. et al.	2024	Metal contaminated agroecosystem	Hybrid nanoparticles	Multiple	Review	New insights into environmental application of hybrid nanoparticles
[147]	Wu, L. et al.	2024	Cd-contaminated soil	Biochar-supported nHAP	Cd	Pepper growth study	Biochar-supported nHAP reduces soil available Cd by 56.8%
[148]	Wu, W. et al.	2025	Zinc pollution in smelting site	nHAP-modified cyanobacterial biochar	Zn	Restoration study	Restoration of zinc pollution using nHAP-modified biochar
[149]	Yuanyuan, L. et al.	2026	Global	Sustainability assessment	Multiple	Systematic review	Global systematic review of sustainability assessment for contaminated site remediation
[150]	Azuazu, I. N. et al.	2023	Niger Delta	Low-carbon remediation	Multiple	Challenges and opportunities	Challenges and opportunities for low-carbon remediation

[151]	Masud, M. A. A. et al.	2023	Global	Biochar for green remediation	Multiple	Review	Critical review of sustainable application of biochar for green remediation
[152]	Alshehri, K. et al.	2023	Global	Nature-based solutions	Multiple	Life cycle assessment	Life cycle assessment and cost-benefit analysis of nature-based solutions

Note: This table includes the 152 references formally cited in the main manuscript.
